# Rabbit Microbiota Changes Throughout the Intestinal Tract

**DOI:** 10.3389/fmicb.2018.02144

**Published:** 2018-09-13

**Authors:** María Velasco-Galilea, Miriam Piles, Marc Viñas, Oriol Rafel, Olga González-Rodríguez, Miriam Guivernau, Juan P. Sánchez

**Affiliations:** ^1^Animal Breeding and Genetics, Institute for Food and Agriculture Research and Technology (IRTA), Barcelona, Spain; ^2^Integral Management of Organic Waste, Institute for Food and Agriculture Research and Technology (IRTA), Barcelona, Spain

**Keywords:** gut microbiota, fecal microbiota, cecal microbiota, feed restriction, meat rabbit, paired analysis, multivariate approaches, 16S Illumina sequencing

## Abstract

To gain insight into the importance of carefully selecting the sampling area for intestinal microbiota studies, cecal and fecal microbial communities of Caldes meat rabbit were characterized. The animals involved in the study were divided in two groups according to the feed intake level they received during the fattening period; *ad libitum* (*n* = 10) or restricted to 75% of *ad libitum* intake (*n* = 11). Cecum and internal hard feces were sampled from sacrificed animals. Assessment of bacterial and archaeal populations was performed by means of Illumina sequencing of 16S rRNA gene amplicons in a MiSeq platform. A total of 596 operational taxonomic units (OTUs) were detected using QIIME software. Taxonomic assignment revealed that microbial diversity was dominated by phyla Firmicutes (76.42%), Tenericutes (7.83%), and Bacteroidetes (7.42%); kingdom Archaea was presented at low percentage (0.61%). No significant differences were detected between sampling origins in microbial diversity or richness assessed using two alpha-diversity indexes: Shannon and the observed number of OTUs. However, the analysis of variance at genus level revealed a higher presence of genera *Clostridium, Anaerofustis, Blautia, Akkermansia, rc4-4*, and *Bacteroides* in cecal samples. By contrast, genera *Oscillospira* and *Coprococcus* were found to be overrepresented in feces, suggesting that bacterial species of these genera would act as fermenters at the end of feed digestion process. At the lowest taxonomic level, 83 and 97 OTUs in feces and cecum, respectively, were differentially represented. Multivariate statistical assessment revealed that sparse partial least squares discriminant analysis (sPLS-DA) was the best approach for this purpose. Interestingly, the majority of the most discriminative OTUs selected by sPLS-DA were found to be differentially represented between sampling origins in univariate analysis. Our study provides evidence that the choice of intestinal sampling area is relevant due to important differences in some taxa’s relative abundance that have been revealed between rabbits’ cecal and fecal microbiota. An appropriate sampling intestinal area should be chosen in each microbiota assessment.

## Introduction

Microbial populations that inhabit animals’ gastrointestinal tract constitute their microbiota: a complex ecosystem, able to autoregulate its own homeostasis. It is well known that a mammal’s intestinal microbiome plays a very important role in metabolic, nutritional, physiological, and immunological processes (Flint et al., 2012) but also in farm animal’s productivity (Heinrichs and Lesmeister, 2005; Drouilhet et al., 2016). A symbiotic relationship is therefore established between the host and its intestinal microbiota. The emergence of next generation sequencing (NGS) techniques together with an increasing reliability of reference taxonomic databases such as SILVA (Yilmaz et al., 2013), RDP (Wang et al., 2007), or Greengenes (McDonald et al., 2012) have allowed a deeper knowledge of the influence that intestinal microbiome exerts on host animals.

In the case of the rabbit, the physicochemical properties of its gastrointestinal tract (near neutral pH, high humidity and stable temperature around 35–40°C) promote the rapid growth of mutualistic microbiota while the animal gets the bacterial fermentation end-products of some materials that cannot be degraded by the host on its own (Mackie, 2002). In these conditions, rabbit intestinal microbiota contains 100–1000 billions of microorganisms per gram covering over 1000 different species, predominating kingdom Bacteria over archaeal populations (Combes et al., 2011). Despite the demonstrated existence of active microbial populations in proximal and distal segments of rabbit gastrointestinal tract (Gouet and Fonty, 1979), cecum is the main fermenter organ. For this reason, most studies that aimed to unravel rabbit’s intestinal microbiota have been focused on the characterization of cecal microbial communities (Abecia et al., 2005; Kušar and Avguštin, 2010; Bäuerl et al., 2014). Cecal microbiota of rabbit and other lagomorph species is dominated by phylum Firmicutes while cecal microbiota of rodents, a relatively close mammalian order, is dominated by phylum Bacteroidetes (Li et al., 2017).

In other monogastric livestock species, such as chicken and pig, previous studies have characterized the differences between their cecal and fecal microbiotas (He et al., 2016; Oakley and Kogut, 2016; Fang et al., 2017). Crowley et al. (2017) compared the microbial composition from different organs of the digestive tract (stomach, jejunum, cecum, appendicular cecum, proximal colon, distal colon, and rectum) in wild rabbits and they found that the different physicochemical properties of each compartment restrict or promote the growth of specific microbial populations. However, little is known about the differences in the composition of the microbial communities that inhabit the domesticated rabbit cecum and feces.

The objective of this study was to characterize and compare the microbial communities of hard feces and cecum content collected from two groups of animals from a meat rabbit line fed with different intake levels. Our results will help establish whether feces could be considered a proxy indicator to assess composition and diversity of intestinal microbiota. This will be particularly important for those studies that require a monitoring of the microbiota over time in order to avoid the manipulation of the animal’s gastrointestinal tract that could alter its microbial composition.

## Materials and Methods

### Experimental Design and Sampling

The sampling materials from animals used in this study came from an experiment conducted at the Institute for Food and Agriculture Research and Technology (IRTA) between July 2012 and July 2014. This experiment was developed to estimate the effect of the interaction between the genotype and the feeding regime on growth, feed efficiency, carcass characteristics, and health status of the animals. Toward this aim, 7,864 animals from Caldes line (Gómez et al., 2002), selected since the 1980’s to increase the average daily gain during the fattening period (32–66 days of age), were controlled since weaning. Animals were housed in 969 collective cages, with a surface of 0.38 m^2^, containing eight rabbits each one. All animals in this experiment were bred under the same management conditions and fed with the same standard pellet diet supplemented with antibiotics (oxytetracycline, valnemulin, and colistin), except during the last fattening week, when an antibiotic free food was provided. During the 5 weeks that the fattening period lasted, food was supplied once per day in a feeder with three places. Details of food composition can be found in **Table 1**. Water was also provided *ad libitum* during the whole fattening period.

**Table 1:** Feed composition on a wet basis.

Component	Amount
Crude fiber (%)	18.70
Crude protein (%)	15.02
Ashes (%)	8.97
Ether extract (%)	3.28
Oxytetracycline (mg/kg)	400
Valnemulin (mg/kg)	30
Colistin (mg/kg)	100

*The average daily feed intake in one *ad libitum* cage was 0.17 kg/day/rabbit which implies 66.48 mg/rabbit of oxytetracycline, 4.99 mg/rabbit of valnemulin and 16.62 mg/rabbit of colistin. The average daily feed intake in one restricted cage was 0.13 kg/day/rabbit which implies 49.86 mg/rabbit of oxytetracycline, 3.74 mg/rabbit of valnemulin and 12.47 mg/rabbit of colistin*.

The animals were under two different feeding regimes: (1) *ad libitum* (V) or (2) restricted (R) feeding to 75% of the *ad libitum* feed intake. The amount of food provided to the animals under R feeding regime in a given week for each batch was obtained as 0.75 times the average feed intake of kits on V from the same batch during the previous week, plus 10% corresponding to the estimated increase of feed intake as the animal grows.

Kits were randomly assigned to one of these two feeding regimes after weaning (32 days of age). They were categorized into two groups according to their size (“big” if body weight at weaning was greater than 700 g or “small” otherwise) in order to obtain homogenous groups regarding animal size within each feeding regime. A maximum of two kits of the same litter were assigned to the same cage, aiming to remove the possible association between cage and maternal effects on animal growth during the fattening period.

For this particular study 23 rabbits from the aforementioned experiment were randomly selected. Their distribution across the different levels of factors is shown in **Table 2**.

**Table 2:** Distribution of animals in groups according to feeding regime and size.

Feeding regime	^a^Size	Number of animals
Restricted	Small	4
	Big	9
*Ad libitum*	Small	1
	Big	9

*^*a*^Animals classified according to their size at weaning: “big” if body weight was greater than 700 g or “small” otherwise*.

At slaughtering (66 days of age) hard feces and cecum samples were collected from each animal, kept cold in the laboratory (4°C) and immediately stored at -80°C until total genomic DNA extraction.

### DNA Extraction, Library Generation and Sequencing

The extraction of total genomic DNA was performed by means of a bead-beating protocol (kit ZR Soil Microbe DNA MiniPrep^TM^-ZymoResearch, Freiburg, Germany) following manufacturer’s recommendations. A total of 250 mg of each cecal and fecal samples was submitted to a mechanical lysis in a FastPrep-24^TM^ Homogenizer (MP Biomedicals, LLC, Santa Ana, CA, United States) at a speed of 1 × 6 m/s for 60 s allowing an efficient lysis of archaea and Gram-positive and negative bacteria species. Purity and integrity of total DNA from each sample was checked in a Nanodrop ND-1000 spectrophotometer equipment (NanoDrop products; Wilmington, DE, United States) following the protocol described by Desjardins and Conklin (2010). All extracts had a proper purity (absorbance ratio 260 nm/280 nm >1.6) to avoid polymerase chain reaction (PCR) inhibition issues during downstream PCR and sequencing steps.

The V4-V5 hypervariable region of total genomic DNA was amplified with specific primers and then re-amplified in a limited-cycle PCR reaction to add sequencing adaptors and 8 nt dual-indexed barcodes of multiplex Nextera^®^ XT kit (Illumina, Inc., San Diego CA, United States) according to manufacturer’s instructions. The initial PCR reactions were performed for each sample (23 cecal and 23 fecal) using 12.5 μl of 2x KAPA HiFi HotStart Ready Mix, 5 μl of each PCR primer: forward universal primer 515Y (5^′^-GTGYCAGCMGCCGCGGTAA-3^′^) and reverse universal primer 926: (5^′^-CCGYCAATTYMTTTRAGTTT-3^′^) (Parada et al., 2016) and 2.5 μl of microbial DNA (5 ng/μl). The initial thermal cycling procedure consisted of an initial denaturation step at 95°C for 3 min, followed by 25 cycles of 95°C for 30 s, 55°C for 30 s and 72°C for 30 s, and a final extension of 72°C for 5 min. The second thermal cycling procedure added the indexes and sequencing adaptors to both ends of the amplified regions by using 25 μl of 2x KAPA HiFi HotStart Ready Mix, 5 μl of each index (i7 and i5), 10 μl of PCR Grade water and 5 μl of the first PCR product. The procedure consisted of an initial denaturation step at 95°C for 3 min, followed by eight cycles of 95°C for 30 s, 55°C for 30 s and 72°C for 30 s, and a final extension of 72°C for 5 min. Final libraries were cleaned up with AMPure XP beads, validated by running 1 μl of a 1:50 dilution on a Bioanalyzer DNA 1000 chip (Agilent Technologies, Inc., Santa Clara, CA, United States) to verify its size, quantified by fluorometry with PicoGreen dsDNA quantification kit (Invitrogen, Life Technologies, Carlsbad, CA, United States), pooled at equimolar concentrations and paired-end sequenced in parallel in a MiSeq Illumina 2 × 250 platform at the Genomics and Bioinformatics Service (SGB) of the Autonomous University of Barcelona.

### Bioinformatics – Sequence Processing

The resulting paired-ended V4–V5 16S rRNA gene reads were assembled into contigs with the python script *multiple_join_paired_ends.py* by using QIIME software (version 1.9.0) (Caporaso et al., 2010). Then the contigs were curated using the QIIME script *split_libraries.py* with default parameters in order to assign contigs to samples and to remove low-quality (Q19 was the minimum acceptable quality score) contigs. UCHIME algorithm (Edgar et al., 2011) was used to remove chimeric sequences generated during the process of DNA amplification. The totality of filtered contigs were clustered into operational taxonomic units (OTUs) with a 97% similarity threshold using the QIIME script *pick_open_reference_otus.py* with default parameters (Rideout et al., 2014) that grouped, through UCLUST algorithm (Edgar, 2010), sequences against Greengenes reference database (version gg_13_5_otus) and also made a *de novo* clustering of those that did not match the database. The generated OTU table was filtered at: (1) sample level by discarding samples with less than 5,000 final contigs and at (2) OTU level by removing OTUs with less than 0.01% counts across samples. Finally, OTU table was normalized using the Cumulative Sum Scaling (CSS) method proposed by Paulson et al. (2013) yielding the normalized abundances of 596 OTUs for 43 samples. Note that three samples (cecal and fecal collected from one rabbit of size class “big” fed under restriction and cecal from another rabbit also of size class “big” and fed under restriction) did not pass the established threshold defined during the edition and quality control processes. In addition to this, in order to always keep parity between samples, i.e., for each animal to have both cecal and fecal samples, one fecal sample (from a rabbit of size class “big” fed under restriction) passing quality control was finally discarded for the next statistical analyses. Therefore, final analyses comprised of both types of samples (hard feces and cecum) from 21 animals. Taxonomic assignment of representative sequences of each OTU defined (596) was conducted by mapping them to the Greengenes reference database gg_13_5_otus with the UCLUST consensus taxonomy assigner (QIIME default parameters). The raw sequence data were deposited in the sequence read archive of NCBI under accession number (SRP149070).

### Statistical Analysis

#### Alpha-Diversity and Univariate Statistical Analysis

In order to compare diversity and richness between fecal and cecal communities, the Shannon and the observed number of OTUs indexes were computed after OTUs rarification at 15,000 contigs. The statistical method used for the communities’ comparison was a paired samples analysis of variance that included the following factors: sampling origin (feces/cecum), feeding regime (*ad libitum*/restricted), the interaction between them and the animal from which the samples were collected. The significance threshold was set at 0.05 type I error.

Differences in OTUs composition between cecal and fecal samples were estimated for those OTUs detected in at least 5% of the samples. For this purpose, analysis of variance were implemented by fitting a model defined by the factors sampling origin (feces/cecum), feeding regime (*ad libitum*/restricted) and the animal from which the samples were collected. Consideration of the animal effect into the model allowed for accounting for the paired structure of the data. The effect of the sampling origin was assessed as the differences between the expected OTUs counts in both cecum and feces. Significance of the sampling origin was based on the F statistic, but instead of defining the threshold for declaring significance based on the theoretical F distribution, empirical bootstrap p-values were computed after 1,000 resamples. The use of bootstrapping allowed inferences to be made from the results obtained without the need for assuming that data are normally distributed. In this case the *p*-value was defined as the proportion of bootstrap rounds showing an F statistic value equal or greater than that obtained with the original data set. *P*-values were corrected defining a false discovery rate (FDR) of 0.05 (Benjamini and Hochberg, 1995).

This bootstrap analysis of variance approach was also implemented to study the effect of the sampling origin on the relative abundance of bacteria at phylum and genus levels.

#### Multivariate Statistical Analysis

In addition to the univariate paired analysis of variance, three multivariate analyses were performed to assess whether there were differences between cecal and fecal communities as a whole, taking into account the dependency between OTUs. The first one was a descriptive analysis using principal coordinate analysis (PCoA) (Gower, 1966) on weighted Unifrac phylogenetic distance matrix (Lozupone and Knight, 2005). The second analysis was also a descriptive technique, principal component analysis (PCA) (Hotelling, 1933), but it was performed considering the paired structure of the data (Liquet et al., 2012). This was achieved by subtracting from the OTU count of a given sample the mean of the two samples belonging to the animal from which they were taken. The last multivariate method implemented was the sparse partial least squares discriminant analysis (sPLS-DA) which is a method based on partial least squares regression applied for classification. PLS consists in a multivariate regression which allows for the correlation of the information contained in a predicting matrix to the information contained in a response matrix or vector (Burnham et al., 1996). In this case, the response was a vector which encoded the sampling origin that we aimed to predict from OTUs content. Moreover, sPLS includes a LASSO penalization to select the most informative predictors. sPLS-DA can simultaneously find, by maximizing the covariance between the predicting and the response matrices, the combination of OTUs which best discriminate samples according to their sampling origin and integrate both data sets in a one-step procedure (Lê Cao et al., 2008). In order to account for individual variation in the data, OTUs content was defined as deviations from individual means, as it was done for PCA. Unlike PCoA or PCA, sPLS-DA is not only a descriptive approach since it can infer which OTUs should be selected to perform the best discrimination of samples according to a given factor; the sampling origin in this study.

R packages “phyloseq,” “mixOmics,” and “ggplot2” were employed for statistical analysis and plotting as elsewhere described (Wickham, 2010; McMurdie and Holmes, 2013; Le Cao et al., 2016).

## Results

### Sequencing and Processing

The sequencing process generated a total of 5,337,066 reads which, after different filtering steps and chimera removal, resulted in a total of 1,707,620 valid contigs. These final sequences were clustered into 596 non-singleton containing OTUs. Each sample had on average 40,657 final contigs (range: 16,415–68,080) and 482 OTUs (range: 411–541) (**Supplementary Table S1**).

### Differences in Diversity and Richness Between Sampling Origins

**FIGURE 1:**
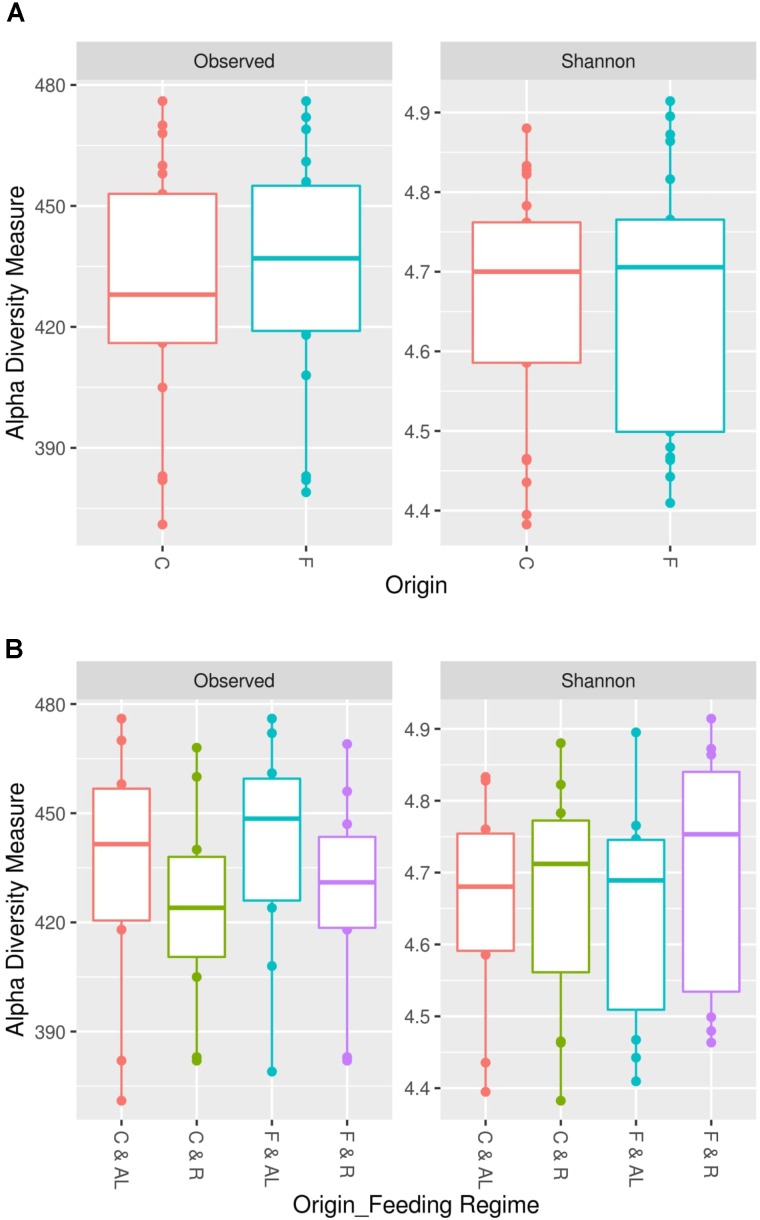
Microbial richness and diversity between cecum and feces samples. The intestinal microbial richness was estimated by the observed number of OTUs index and the microbial diversity was studied by Shannon index. Both indexes were computed at 15,000 contigs. **(A)** Significant differences in microbial richness and diversity between cecum (C) and feces (F) samples were not identified (*P* > 0.05; paired samples analysis of variance). **(B)** Significant differences in microbial richness and diversity between cecum and feces samples in *ad libitum* (AL) or restricted (R) rabbits were not identified (*P* > 0.05; paired samples analysis of variance).

In this study, we found an average of 428 observed OTUs in cecum samples and 433 in feces samples. The estimated Shannon indexes were 4.66 and 4.67 in cecum and feces samples, respectively (**Table 3**). The comparison of alpha diversities between fecal and cecal samples did not reveal any significant difference in microbial diversity or richness at 15,000 contigs rarification (**Figure 1A**, *P* > 0.05) nor when both sampling origins were compared within feeding regime (**Figure 1B** and **Table 3**, *P* > 0.05). In contrast, the observed number of OTUs index showed significant differences between feeding regimes as the means estimated were 425 in restricted animals and 437 in *ad libitum* animals (**Table 3**, *P* = 0.03, *p*-value is not shown in table nor figures).

**Table 3:** Estimated mean and standard deviation of observed number of OTUs and Shannon α-diversity indexes calculated in cecum and feces samples.

Feeding regime	Index	Cecum samples	Feces samples	*P*
Restricted	Observed OTUs	423.91 (27.40)	427.91 (27.19)	0.73
	Shannon	4.67 (0.16)	4.69 (0.17)	0.70
*Ad libitum*	Observed OTUs	433.90 (35.08)	440.40 (30.25)	0.66
	Shannon	4.65 (0.15)	4.64 (0.16)	0.93
Average	Observed OTUs	428.67 (30.91)	433.86 (28.67)	0.58
	Shannon	4.66 (0.15)	4.67 (0.16)	0.81

### Taxonomic Characterization of Cecum and Feces Microbial Communities

The final OTU table encompassed 596 OTUs of which 307 were annotated in Greengenes database gg_13_5_otus and 289 corresponded to new reference OTUs constructed from a random sampling of sequences that did not map against the reference. Five hundred and eighty out of the 596 declared OTUs could be taxonomically assigned at kingdom level. All of them could be assigned at phylum and class levels, belonging to 8 and 12 different taxa, respectively. Five hundred and seventy seven OTUs could be assigned at order level to 13 different taxa. At family level, 308 OTUs could be assigned to 22 different taxa. One hundred and eighteen OTUs could be assigned at genus level to 23 different taxa while only 10 OTUs were taxonomically assigned at species level. It is important to stress that resolution of MiSeq technology in this study impaired taxonomic assignment capacity at family level since it was only possible in 51% of OTUs and, more drastically, at genus level allowing the assignment of only 20% of them. Nevertheless, given the large importance of functional roles played by bacteria that can be assigned at genus level, the analysis of differential representation of genera between the two sampling origins was conducted for those in which taxonomic assignment at this level was possible.

The two types of samples showed similar relative abundances for taxa and the predominant phyla were, in both cases, Firmicutes (present in an average percentage of 76.28 in feces and 76.55 in cecum), followed by Tenericutes (8.17 in feces and 7.48 in cecum), and Bacteroidetes (7.37 in feces and 7.46 in cecum) (**Table 4**). In spite of the small magnitude of the differences they reached significance in some cases (*P* < 0.05). As it can be observed in **Table 4**, phyla Actinobacteria and Verrucomicrobia were found to be overrepresented in cecum samples, while Cyanobacteria and Tenericutes were overrepresented in feces. The only phylum belonging to kingdom Archaea that could be identified was Euryarchaeota which was presented in an average percentage of 0.61‰ in both sampling origins. All species of this phylum were taxonomically assigned to the methanogenic genus *Methanobrevibacter*.

**Table 4:** Microbial composition at phylum level in cecum and feces.

Phylum	Mean relative abundance in cecum (%) (SD)	Mean relative abundance in feces (%) (SD)	Difference cecum-feces ± SE	*P_FDR_*
Actinobacteria	0.729 (0.097)	0.617 (0.119)	0.110 ± 0.023	0.000
Bacteroidetes	7.458 (1.243)	7.367 (1.263)	0.092 ± 0.090	0.473
Cyanobacteria	0.873 (0.440)	1.399 (0.670)	-0.514 ± 0.072	0.000
Euryarchaeota	0.061 (0.096)	0.062 (0.095)	-0.001 ± 0.011	0.928
Firmicutes	76.546 (1.733)	76.276 (1.809)	0.253 ± 0.170	0.215
Proteobacteria	1.613 (0.363)	1.634 (0.312)	-0.016 ± 0.043	0.783
Tenericutes	7.484 (0.899)	8.172 (1.057)	-0.681 ± 0.169	0.000
Verrucomicrobia	1.810 (0.378)	1.651 (0.300)	0.158 ± 0.034	0.000
Unknown	3.427 (0.433)	2.822 (0.674)	0.599 ± 0.092	0.000

The predominant classes in both sampling origins were *Clostridia* (76.14%), *Mollicutes* (7.54%), and *Bacteroidia* (7.41%). At family level, the predominant taxa were *Ruminococcaceae* (44.37%) and *Lachnospiraceae* (36.51%) both belonging to phylum Firmicutes. Finally, results contained in **Table 5** show that predominant genera were *Ruminococcus* (5.13%), *Oscillospira* (2.47%), *Bacteroides* (2.40%), and *Blautia* (2.31%). Paired samples analysis of variance implemented to study the effect of the sampling origin on the relative abundance of species at genus level revealed that eight genera, out of the 23 in which taxonomic assignment was possible, were differentially represented between feces and cecum. Genera *Clostridium, Anaerofustis, Blautia, Akkermansia, rc4-4*, and *Bacteroides* were overrepresented in cecum while feces showed a higher relative abundance of genera *Oscillospira* and *Coprococcus* (**Table 5**). Genera *Anaerofustis* and *rc4-4* showed the smallest relative abundances (0.14 and 0.19%, respectively) while the rest of the genera ranged between 1.21 and 2.48%.

**Table 5:** Microbial composition at genus level, grouped by phylum, in cecum and feces.

Genus	Mean relative abundance in cecum (%) (SD)	Mean relative abundance in feces (%) (SD)	Difference cecum-feces ± SE	*P_FDR_*
**Actinobacteria**				
*Adlercreutzia*	0.175 (0.038)	0.149 (0.043)	0.023 ± 0.012	0.092
**Bacteroidetes**				
*Bacteroides*	2.436 (0.571)	2.358 (0.562)	0.079 ± 0.028	0.023
*Butyricimonas*	0.160 (0.173)	0.158 (0.163)	0.001 ± 0.013	0.959
*Odoribacter*	0.164 (0.091)	0.166 (0.077)	-0.001 ± 0.011	0.959
*Parabacteroides*	0.212 (0.217)	0.204 (0.210)	0.008 ± 0.006	0.233
*Rikenella*	0.475 (0.264)	0.457 (0.261)	0.020 ± 0.020	0.421
**Euryarchaeota**				
*Methanobrevibacter*	0.061 (0.096)	0.061 (0.095)	-0.001 ± 0.011	0.959
**Firmicutes**				
*Anaerofustis*	0.148 (0.070)	0.124 (0.057)	0.024 ± 0.008	0.024
*Anaerostipes*	0.302 (0.141)	0.360 (0.137)	-0.059 ± 0.028	0.083
*Blautia*	2.532 (0.351)	2.086 (0.285)	0.444 ± 0.058	0.000
*Clostridium*	1.585 (0.221)	1.437 (0.221)	0.148 ± 0.029	0.000
*Coprobacillus*	0.173 (0.113)	0.164 (0.119)	0.009 ± 0.014	0.583
*Coprococcus*	1.163 (0.300)	1.295 (0.318)	-0.130 ± 0.028	0.000
*Epulopiscium*	0.210 (0.130)	0.194 (0.114)	0.017 ± 0.027	0.583
*Oscillospira*	2.345 (0.420)	2.598 (0.355)	-0.255 ± 0.058	0.000
*Phascolarctobacterium*	0.307 (0.240)	0.311 (0.248)	-0.007 ± 0.034	0.959
*rc4-4*	0.198 (0.040)	0.173 (0.043)	0.026 ± 0.010	0.034
*Roseburia*	0.056 (0.072)	0.078 (0.069)	-0.022 ± 0.012	0.123
*Ruminococcus*	5.070 (0.736)	5.197 (0.814)	-0.124 ± 0.091	0.233
**Proteobacteria**				
*Desulfovibrio*	0.507 (0.140)	0.493 (0.114)	0.014 ± 0.013	0.390
*Oxalobacter*	0.125 (0.067)	0.104 (0.070)	0.022 ± 0.011	0.083
**Tenericutes**				
*Anaeroplasma*	0.263 (0.162)	0.229 (0.162)	0.033 ± 0.014	0.054
**Verrucomicrobia**				
*Akkermansia*	1.810 (0.378)	1.651 (0.300)	0.158 ± 0.034	0.000
**Unknown**	79.523 (1.509)	79.954 (1.461)	0.599 ± 0.092	0.000

Paired bootstrap analysis of variance revealed that 180 OTUs showed abundances significantly different between sampling origins: 83 and 97 OTUs were overrepresented in fecal and cecal samples, respectively (**Supplementary Table S2** and **Table 6**, this last shows the 10 OTUs most differentially represented between both types of samples). In fecal samples these 83 overrepresented OTUs were assigned, at the lowest taxonomic level, to the candidate species *Eutactus* (1 OTU) and *Flavefaciens* (2 OTUs); candidate genera *Coprococcus* (3 OTUs), *Oscillospira* (7 OTUs) and *Ruminococcus* (3 OTUs); candidate families *Ruminococcaceae* (10 OTUs) and *S24-7* (4 OTUs); candidate orders *Clostridiales* (34 OTUs), *RF32* (1 OTU), *RF39* (10 OTUs), and *YS2* (7 OTUs); and candidate class *Alphaproteobacteria* (1 OTU). On the other hand, the 97 OTUs overrepresented in cecal samples were assigned to the candidate genera *Akkermansia* (4 OTUs), *Anaerofustis* (1 OTUs), *Blautia* (10 OTUs), *Clostridium* (4 OTUs), *Oscillospira* (1 OTU), *Phascolarctobacterium* (1 OTU), and *Ruminococcus* (2 OTUs); candidate families *Mogibacteriaceae* (2 OTUs), *Christensenellaceae* (1 OTU), *Clostridiaceae* (1 OTU), *Coriobacteriaceae* (2 OTUs), *Lachnospiraceae* (16 OTUs), *Rikenellaceae* (1 OTU), and *Ruminococcaceae* (8 OTUs); candidate orders *Bacteroidales* (1 OTU), *Clostridiales* (30 OTUs), and *ML615J-28* (1 OTU); and candidate class *Betaproteobacteria* (1 OTU) while 10 OTUs could not be assigned to any taxonomic level. These results at OTU level show remarkable coincidences with the analyses directly performed on the relative abundance of taxa at phylum and genera levels. This is consistent with two possibilities: a case of phylum encompassing one or a reduced number of genera (like *Verrucomicrobia* and *Akkermansia*), or when all the OTUs in a given taxa show an effect on the same direction (for example an overrepresentation of the 10 OTUs assigned to genus *Blautia* in cecal samples).

**Table 6:** OTUs most differentially represented between fecal and cecal samples.

OUT ID and taxonomical assignment	Mean abundance	Difference	*P_FDR_*	^a^Discriminant
	(CSS OTU units) (SD)	Cecum-feces ± SE		sPLS-DA
**NR57**, Firmicutes; *Clostridia*; *Clostridiales*; *Ruminococcaceae*	3.603 (2.298)	-2.503 ± 0.311	0.000	NO
**NR60**, Firmicutes; *Clostridia*; *Clostridiales*	2.944 (2.283)	-2.247 ± 0.201	0.000	NO
**581388**, Cyanobacteria; *4C0d-2*; *YS2*	3.847 (1.935)	-2.034 ± 0.173	0.000	YES
**NR28**, Firmicutes; *Clostridia*; *Clostridiales*; *Ruminococcaceae*	7.464 (1.893)	-1.860 ± 0.180	0.000	YES
**550894**, Cyanobacteria; *4C0d-2*; *YS2*	3.651 (1.871)	-1.713 ± 0.219	0.000	YES
**NR12**, Firmicutes; *Clostridia*; *Clostridiales*	4.650 (1.058)	1.706 ± 0.125	0.000	YES
**589410**, Cyanobacteria; *4C0d-2*; *YS2*	1.649 (1.468)	-1.544 ± 0.190	0.000	YES
**542830**, Cyanobacteria; *4C0d-2*; *YS2*	2.340 (1.800)	-1.313 ± 0.309	0.011	NO
**NR411**, Proteobacteria; *Alphaproteobacteria*; *RF32*	1.990 (1.551)	-1.214 ± 0.255	0.000	YES
**197832**, Firmicutes; *Clostridia*; *Clostridiales*; *Lachnospiraceae*; *Coprococcus*	3.295 (3.191)	-1.208 ± 0.357	0.011	NO

*^*a*^Last column indicates whether the OTU belongs to component 1 of sPLS-DA*.

### Clusterization of Samples According to Their Origin With Different Multivariate Methods

**FIGURE 2:**
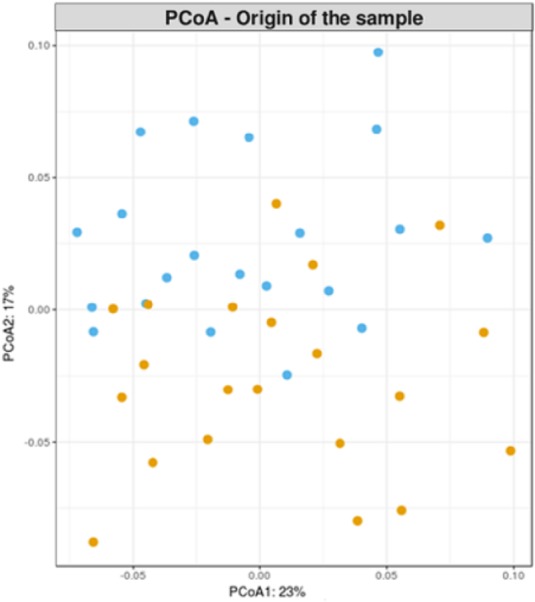
Principal coordinate analysis on weighted Unifrac phylogenetic distance matrix. Cecal and fecal samples are blue and orange colored, respectively.

First, a principal coordinate analyses (PCoA) on weighted Unifrac phylogenetic distance matrix calculated from the final OTU table was performed. In **Figure 2**, each sample is located in a specific position of a bidimensional chart in function of its microbial composition. No clear pattern of separation of samples by their sampling origin could be appreciated.

**FIGURE 3:**
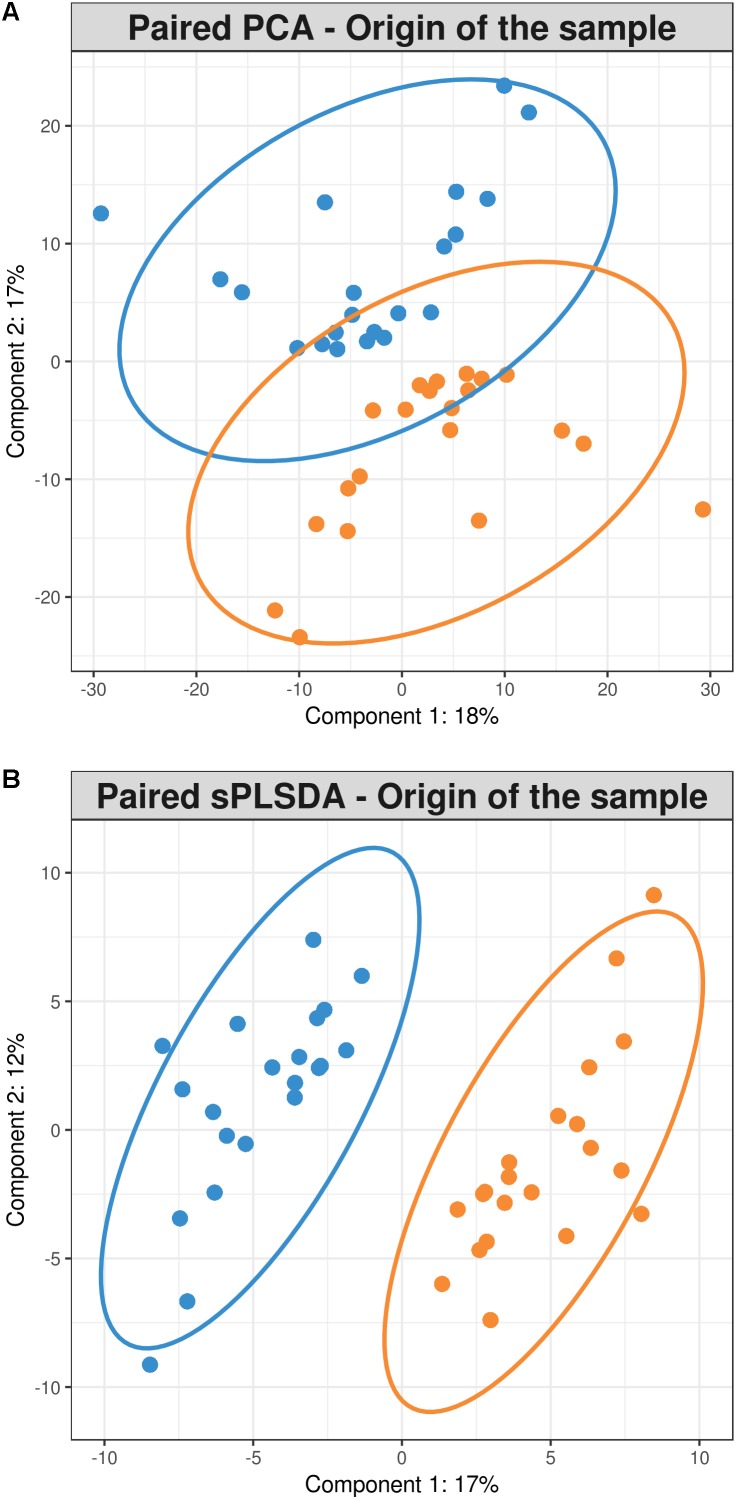
**(A)** Paired samples principal component analysis **(B)** Paired samples sparse partial least squares discriminant analysis representing 21 cecum (blue) and 21 feces (orange) samples.

The paired PCA was implemented in order to take into account the fact that each pair of cecal and fecal samples which belonged to the same rabbit showed a better separation pattern than PCoA. Components 1 and 2 explained 18 and 17% of variance, respectively (**Figure 3A**).

**FIGURE 4:**
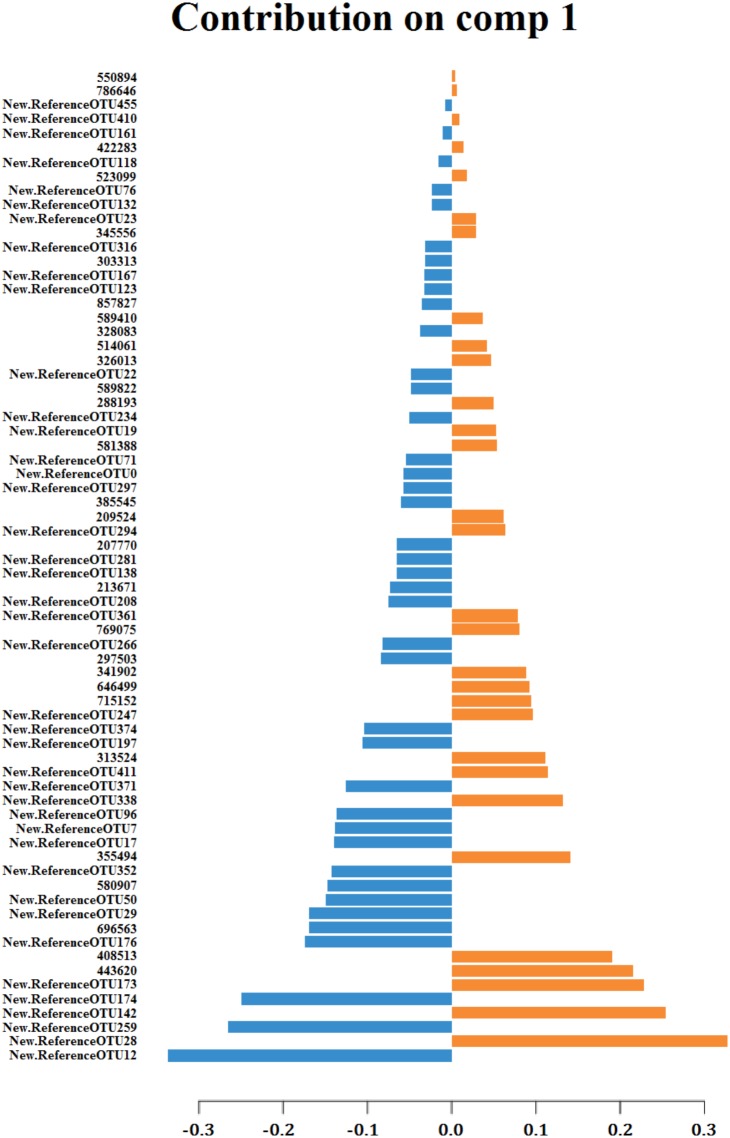
Contribution of each OTU, on component 1 of sPLS-DA, to the discrimination of samples regarding to their sampling origin: cecum (blue) or feces (orange).

But the multivariate method that best clustered the samples according to their sampling origin was the paired sPLS-DA which took into account the fact that two different samples were collected from the same animal and indeed it was only conducted with the OTUs that best discriminated samples by their sampling origin (70 and 50 for components 1 and 2, respectively) (**Figure 3B**). The 70 OTUs that were part of the component 1 explained 17% of total variance. Forty of them were found to be overrepresented in cecum and 30 in feces (**Figure 4**). It should be noted that 66 OTUs declared as differentially represented between cecum and feces by sPLS-DA were also declared as differentially represented between sampling origins by the univariate bootstrap analyses of variance previously performed. The 10 OTUs most differentially represented between sampling origins (according to univariate analyses of variance) can be found in **Table 6** with an indication of whether the OTU belonged to the first component of the sPLS-DA analysis. The representative sequences of these OTUs are showed in **Supplementary Data Sheet 1**.

## Discussion

In this study, we aimed to evaluate the importance of selecting a proper sampling intestinal area for rabbit microbiota studies. To give an answer to this question, 16S rRNA gene amplicons from cecal and fecal samples collected from 21 meat rabbits randomly distributed in two feeding groups with different intake levels were sequenced in an Illumina MiSeq platform.

Similar to our work, sequencing results from other studies performed on gastrointestinal microbial populations of caecotrophagic animals (rabbit and guinea pig) (Zeng et al., 2015; Crowley et al., 2017), hare and pika (which, like rabbit, are members of the clade Glires) (Li et al., 2017), other livestock species like broiler chicken (Han et al., 2016) and on other environments, such as goats’ rumen (Wang et al., 2016) or sheeps’ lung (Glendinning et al., 2016), showed variable results in the average number of final contigs per sample. Our results are in accordance with the well-known fact that sequencing of the 16S rRNA gene could be strongly influenced by different factors like the storage of the sample, the method used for DNA extraction and library generation or the sequencing platform (Pollock et al., 2018). In addition, the variance found in the average final number of contigs and OTUs per sample can be accentuated by the software used, the parameters chosen for sequence filtering or the strategy followed for OTU picking (Allali et al., 2017). As in the present study in which the number of final contigs per sample ranged from 16,415 to 68,080, the study performed by Correa-Fiz et al. (2016) also showed a large variation (ranging from a minimum of 7,338 to a maximum of 844,521 final contigs per sample). On the other hand, the fact that some studies (Zeng et al., 2015; Wang et al., 2016) presented a larger number of OTUs per sample (range: 1,600–6,900) than the present one (range: 411–541) would be due to the fact that they used a different strategy for OTU picking by including an additional de novo clusterization step of sequences which did not match against the reference database.

Our estimates of alpha-diversity with Shannon and the observed number of OTUs indexes did not reveal significant differences between sampling origins. The fact that fecal samples were collected directly from rectum at slaughter could reduce the chances of environmental contamination, which contributed to reduce differences in terms of diversity or richness between sampling origins. Similarly, Zeng et al. (2015) who characterized the cecal and fecal microbiota of two groups of rex rabbits with high or low body weight did not observe differences either in diversity or richness when they compared alpha-diversity indexes between both sampling origins. However, in the study performed by He et al. (2016) in which they compared microbial diversity and richness between cecum and feces samples collected from pigs, they found that fecal samples had a significantly higher alpha-diversity than cecal samples.

With regard to the taxonomic characterization of microbial diversity of cecum and feces, our results are consistent with previous studies on growing rabbit intestinal microbiota (Monteils et al., 2008; Massip et al., 2012; Combes et al., 2017). Nevertheless, relative abundances of the main phyla were different between studies. A quantitative comparison of our study with the first two, shows that they found a higher percentage of Firmicutes (90%) and approximately half the amount of Bacteroidetes (4%). Differences between these phyla could be related to sample storage conditions, as Bahl et al. (2012) demonstrated their importance in Firmicutes to Bacteroidetes 16S rRNA ratio in human fecal samples. Another putative explanation for these discrepancies could be related to updates and changes to the reference databases. For example, it is noteworthy that the presence of phylum Tenericutes was revealed in our study, which had not been reported in previous studies in rabbits. The fact that the only class that phylum Tenericutes contains, *Mollicutes*, was previously classified within phylum Firmicutes is the most plausible hypothesis to explain the differences in the relative abundance of phylum Firmicutes found between our study and previous ones (Monteils et al., 2008; Massip et al., 2012; Combes et al., 2017). The relative abundance of this phylum in our study was situated in the same range as phylum Bacteroidetes. In previous studies it was usual to find phylum Actinobacteria as the third most abundant. Other putative reason for explaining differences could be due to the fact that different 16S rRNA gene regions were sequenced: V3–V4 hypervariable regions in Massip et al. (2012) and Combes et al. (2017), the whole gene in Monteils’ study and V4–V5 hypervariable regions in our study. Another hypothesis could be that the pair of primers employed in our study hybridized better for the sequences belonging to this phylum than the pairs of primers used in previous studies.

Similar to the cecal microbial characterization at class level of rex rabbits performed by Zou et al. (2016) our results revealed that the predominant class was *Clostridia*. But in contrast, they found *Bacteroidia* as the second predominant class while it was the third, followed by *Mollicutes*, according to our results. Our study revealed that the predominant families within phylum Firmicutes were *Ruminococcaceae* and *Lachnospiroceae* in agreement with the results of Massip et al. (2012). As with cecal microbial characterization at genus level of rex rabbits performed by Zou et al. (2016), our results revealed that the predominant genera were *Ruminococcus* and *Oscillospira*. But in contrast, we found *Bacteroides* and *Blautia* to be the following predominant genera while they reported that *Coprococcus* and *Bacteroides* were the next more abundant.

Note that all Archaea species detected in our study belonged to genus *Methanobrevibacter* which encompasses different hydrogenotrophic methane-producing species. The presence of this genus in rumen microbial communities is well known (Henderson et al., 2013; Patra et al., 2017). Moreover, previous studies have also described its presence in the gastrointestinal tract of humans (Thomas et al., 2017) and monogastric animals (Hou et al., 2016; Luo et al., 2017); including rabbit as Kušar and Avguštin (2010) reported in their study. Nonetheless, Mi et al. (2018) revealed the low presence of methanogenic archaea compared to Bacteria domain in rabbit cecum, due to its acidic pH (≈5.8) which does not favor the growth of methanogenic archaea. It is noteworthy to mention that Mi et al. (2018) found *Methanobrevibacter* as the main archaeal population. The small ratio between archaea/bacteria of cecal and fecal samples affected the archaea sequence detection, resulting in a very low archaeal biodiversity.

Although we observed similar microbial diversity and richness between feces and cecum samples, both multivariate and bootstrap univariate analysis revealed that community structures were significantly different in both types of samples. Our results revealed an enrichment of six known genera in cecal samples and two genera in fecal samples considered in detail below.

Despite the fact that the overall relative abundance of phylum Firmicutes did not show differences between sampling origins, most of the genera differentially represented in both types of samples belong to this phylum. This is not surprising because three quarters of bacteria belong to this phylum, which encompasses a large number of lower taxonomic groups. All genera differentially represented within this phylum belong to different families of class *Clostridia*. Genus *Clostridium* (family *Clostridiaceae*) is an anaerobic Gram-positive bacteria whose presence in intestinal microbiota has been reported in human (Lloyd-Price et al., 2016) and many animal species like mouse (Uebanso et al., 2017), chicken (Han et al., 2016; Oakley and Kogut, 2016), or pig (Fang et al., 2017). Bäuerl et al. (2014) reported a greater presence of this genus in cecal microbiota of rabbits affected by epizootic rabbit enteropathy (ERE) than in healthy animals. But not all *Clostridium* species are pathogenic and it is possible to find this genus in normal microbiota as Oakley and Kogut (2016) reported its presence in the cecum of 6-week healthy broiler chickens. Probably, the majoritiy of *Clostridum* species that inhabit rabbit cecum are cellulose-degrading symbiotic microorganisms that help the host in digestion of plant materials. Little is known about the presence of *Anaerofustis* (family *Eubacteriaceae*) in intestinal communities. Arrazuria et al. (2016) found an association between the presence of this genus in cecal samples collected from female rabbits and *Mycobacterium avium* *paratuberculosis* infection. Some *Anaerofustis* species could be involved in the fermentation of carbohydrates and glucose metabolism in the cecum (Lawson, 2015), which could be compatible with the overrepresentation we observed for this genus in cecum which is well known to be the main fermenting organ in rabbits. Within *Ruminococcaceae*, the most abundant family of phylum Firmicutes, the genus *Oscillospira* were overrepresented in fecal samples. This genus has been proved to be one of the core genera of some herbivore’s rumen microbiota like cattle or sheep (Mackie et al., 2003) and horse’s fecal microbiota (O’Donnell et al., 2013). It is a non-cultured anaerobic bacteria but now, thanks to next generation sequencing, we can detect it. Zeng et al. (2015) also reported an overrepresentation of *Oscillospira* in soft feces, which indicates that species of this genus could be involved in fermentation processes as Gophna et al. (2017) inferred that some *Oscillospira* species are butyrate producers. Within the second most abundant family, *Lachnospiraceae*, we found an overrepresentation of genera *Blautia* and *Coprococus* in cecum and feces, respectively. *Blautia* is an important member of animal intestinal microbiota, especially after weaning as Chen et al. (2017) reported in their study with piglets during the weaning transition. Park et al. (2012, 2013) isolated two *Blautia* species in human feces able to ferment carbohydrates and degrade glucose producing acetate and lactate. Consistent with these previous studies and with the one done by Zeng et al. (2015) in which they found a higher representation of *Blautia* in soft feces than in hard feces, the relative enrichment of this genus in cecum versus feces observed in our study could imply that it plays an important role in carbohydrate and glucose digestion in rabbit cecum. On the other hand, *Coprococcus* is an anaerobic Gram-positive bacteria that actively ferments carbohydrates, producing butyric and acetic acids with formic or propionic acids (Holdeman and Moore, 1974). Some studies have previously described the presence of this genus in human (Canani et al., 2016) and horse (Mach et al., 2017) feces. An overrepresentation of *Coprococcus* in rabbit feces could be due to the fact that members of this genus actively participate in fermentation processes in the cecum and after having played their role they cannot be fixed to intestinal walls again and they are expelled with the feces. It is thought that these bacteria found in the final product of feed digestion could be dead bacteria (Fu et al., 2018).

Within the phylum Bacteroidetes, the only genus differentially represented between sampling origins was *Bacteroides* (family *Bacteroidaceae*). *Bacteroides* is an anaerobic Gram-negative bacteria that constitutes an important portion of the mammalian gastrointestinal microbiota (Jandhyala et al., 2015; Rodríguez et al., 2015). This genus has an important role in the degradation of vegetal polysaccharides (Fang et al., 2017) and in amino acid fermentation (Dai et al., 2011) which could be the reason for its overrepresentation in cecum where it is supposed to play an active role.

Finally, *Akkermansia* is also a well-known genus of phylum Verrumicrobia that inhabits intestinal microbiota of mammals (Derrien et al., 2004; Borton et al., 2017) and recently found in reptiles (Rawski et al., 2016; Ouwerkerk et al., 2017). Several studies have demonstrated that some *Akkermansia* species are mucin degraders (Belzer and de Vos, 2012) related with gut inflammation. However, current studies have elucidated that these species also contribute to the reparation of mucosal wounds (Alam et al., 2016) and they could be employed as probiotics (Gómez-Gallego et al., 2016). Previous studies that have characterized microbial communities of different sections across rabbit and chicken gastrointestinal tracts have also found a significant overrepresentation of this genus in cecum with respect to other sections (Zeng et al., 2015; Han et al., 2016). Moreover, Borton et al. (2017) reported an increase in the relative abundance of these bacteria in mouse gut as a consequence of low levels of inflammation. For all of this, we hypothesize that the presence of *Akkermansia* species in cecum could be involved in the formation of a protective mucosa layer that would help rabbits to deal with inflammatory processes.

It is important to note that different studies have identified genera *Bacteroides, Akkermansia*, and *Oscillospira* as obesity-associated intestinal microbial species (de la Cuesta-Zuluaga et al., 2017; Zhang et al., 2017; Zhao et al., 2017) as well as Tan et al. (2018) have found an association between particular species of genera *Akkermansia* and *Clostridium* with psoriasis in humans. We think that careful consideration of the sampling area in this kinds of studies is important to ensure reliable detection of these genera. Monitoring these genera as plausible obesity indicators could be considered in future association studies in order to link intestinal microbiota and particular production traits, such as growth or feed efficiency in livestock animals.

Furthermore, in this study different multivariate approaches to group samples by their origin were performed and different results were obtained due to the fact that the principles on which they are based are different. PCA transformed the 596 potentially correlated variables (OTUs) into a smaller number of uncorrelated variables, or principal components, so that the first component captured as much of the existing variability in the data set. On the contrary, PCoA was based on the Unifrac dissimilarity matrix containing distances between samples in function of their microbial composition in order to represent these phylogenetic distances, with the lowest possible dimensional coordinates. The paired PCA, although it captures the maximum possible variability, did not necessarily capture the part that explains the most important variation according to the categorical variable for which we wanted to classify our samples; the sampling origin in this case (James et al., 2013). According to our results, the approach that best discriminated samples according to their sampling origin was the paired sPLS-DA. It took into account the complex structure of the experimental design in which two different samples were collected from two different “compartments” of the same individual at the same time. This multivariate method allowed for the capture of the sampling origin effect within the animal separately from the variation between animals. Decomposing the within variance from the between variance (Liquet et al., 2012) enables the finding of those OTUs differentially represented between origins which best discriminate both types of samples.

The results of our study show that, overall, the microbial structures of rabbit feces and cecum are similar in terms of richness and diversity, since it should be remembered that we have compared biological samples belonging to locations closely situated throughout the animal intestinal tract that share similar physicochemical conditions. Furthermore, fecal samples were collected from the rectum avoiding the contact of microorganisms with the natural environment and, consequently, with the oxygen that would cause oxidative stress and more drastic changes in some bacterial populations. Nevertheless, it is important to bear in mind the existence of compositional differences in the relative abundance of an important number of taxa and OTUs. Both sampling origins contained the same eight phyla but the relative abundances of half of them were differentially represented between origins. Similarly, at genus level, we found an overrepresentation of some genera such as *Blautia* or *Akkermansia* in cecal samples which would be involved in carbohydrate digestion and in immune protection against inflammation. On the other hand, an overrepresentation of genera *Oscillospira* and *Coprococcus* in fecal samples could indicate an active participation of these bacteria in fermentation at the end of the feed digestion process or correspond to dead species that were excreted once they have played their main role in the cecum. Finally at OTU level we found, with both univariate and multivariate approaches, that 66 were differentially represented between origins in all analyses performed. According to our results, we propose the collection of feces in those studies aiming for a shallow characterization of the intestinal microbiota. On the contrary, for those studies interested in a specific characterization of the composition of microbial communities, it is necessary to consider the fact that important differences in the relative abundance of some taxa, even at phylum level, between cecum and feces have been reported. The decision as to which area of the intestinal tract should be sampled will therefore depend on the objectives of each study.

To sum up, the existence of diversity and compositional differences between rabbit cecum content and internal hard feces microbial communities has been revealed in the present study. In future studies, cecal microbiota of a larger number of rabbits bred under different management conditions, such as feeding regime or the presence of antibiotics in the feed, need to be analyzed to gain insight into the effect of these conditions on rabbit intestinal microbiota and the effect of microbial diversity and composition on animal performance.

## Ethics Statement

This study was carried out in accordance with the recommendations of the animal care and use, committee of the Institute for Food and Agriculture Research and Technology (IRTA). The protocol was approved by the committee of the Institute for Food and Agriculture Research and Technology (IRTA).

## Author Contributions

JS, MP, OR, and MV conceived the experimental design. JS, OR, MP, and MV-G collected biological samples. MV-G, OG-R, MP, and MG processed the samples in the laboratory. MV-G processed and analyzed the sequencing data, interpreted data, prepared figures and tables, and wrote the manuscript. JS and MV helped analyzing the sequencing data. JS, MP, MV, and MG helped interpreting the data, and wrote and revised the manuscript. All authors read and approved the final manuscript.

## Conflict of Interest Statement

The authors declare that the research was conducted in the absence of any commercial or financial relationships that could be construed as a potential conflict of interest.
