# Supplementary material for: Rabbit Microbiota Changes Throughout the Intestinal Tract
Source: Front Microbiol. 2018 Sep 13;9:2144. doi: 10.3389/fmicb.2018.02144 (PMC6146034; doi:10.3389/fmicb.2018.02144)
Supplement: Supplementary file 1 [file Table_1.DOCX]

**TABLE S1| Summary of metadata and OTU tables.**

| **Rabbit ID** | **Feeding Regime** | **Origin** | **No initial sequences** | **No final sequences** | **OTU number** |
| --- | --- | --- | --- | --- | --- |
| 113061 | Ad libitum | Cecum | 189 825 | 66 886 | 458 |
| 113061 | Ad libitum | Feces | 159 674 | 58 402 | 471 |
| 113101 | Ad libitum | Cecum | 173 618 | 58 805 | 517 |
| 113101 | Ad libitum | Feces | 120 847 | 41 672 | 507 |
| 113064 | Restricted | Cecum | 65 274 | 22 024 | 459 |
| 113064 | Restricted | Feces | 110 027 | 38 949 | 482 |
| 113087 | Restricted | Cecum | 119 866 | 38 489 | 514 |
| 113087 | Restricted | Feces | 189 836 | 68 080 | 523 |
| 115804 | Restricted | Cecum | 89 808 | 30 558 | 451 |
| 115804 | Restricted | Feces | 110 928 | 38 186 | 462 |
| 115231 | Restricted | Cecum | 80 883 | 25 563 | 411 |
| 115231 | Restricted | Feces | 157 615 | 41 635 | 422 |
| 115263 | Restricted | Cecum | 62 914 | 20 347 | 414 |
| 115263 | Restricted | Feces | 79 270 | 28 610 | 433 |
| 113210 | Restricted | Cecum | 54 571 | 16 415 | 462 |
| 113210 | Restricted | Feces | 73 208 | 25 905 | 482 |
| 115040 | Ad libitum | Cecum | 125 282 | 42 774 | 485 |
| 115040 | Ad libitum | Feces | 143 182 | 50 473 | 482 |
| 115776 | Restricted | Cecum | 62 347 | 22 035 | 445 |
| 115776 | Restricted | Feces | 113 805 | 32 274 | 469 |
| 113133 | Ad libitum | Cecum | 79 711 | 24 641 | 417 |
| 113133 | Ad libitum | Feces | 106 305 | 31 062 | 424 |
| 113150 | Ad libitum | Cecum | 91 105 | 28 390 | 500 |
| 113150 | Ad libitum | Feces | 85 920 | 27 021 | 489 |
| 115240 | Restricted | Cecum | 91 364 | 22 548 | 468 |
| 115240 | Restricted | Feces | 65 777 | 23 168 | 469 |
| 115162 | Restricted | Cecum | 192 857 | 60 444 | 507 |
| 115162 | Restricted | Feces | 78 863 | 24 470 | 485 |
| 115124 | Ad libitum | Cecum | 159 913 | 49 757 | 488 |
| 115124 | Ad libitum | Feces | 98 913 | 32880 | 486 |
| 115279 | Ad libitum | Cecum | 195 975 | 60 580 | 498 |
| 115279 | Ad libitum | Feces | 156 229 | 50 705 | 510 |
| 115280 | Ad libitum | Cecum | 174 784 | 56 390 | 506 |
| 115280 | Ad libitum | Feces | 148 880 | 47 751 | 523 |
| 115379 | Ad libitum | Cecum | 240 333 | 66 847 | 532 |
| 115379 | Ad libitum | Feces | 201 265 | 57 850 | 541 |
| 113238 | Ad libitum | Cecum | 217 076 | 67 179 | 532 |
| 113238 | Ad libitum | Feces | 186 912 | 60 045 | 526 |
| 113115 | Restricted | Cecum | 156 466 | 43 654 | 479 |
| 113115 | Restricted | Feces | 117 032 | 40 182 | 487 |
| 113198 | Restricted | Cecum | 102 019 | 29 623 | 525 |
| 113198 | Restricted | Feces | 106 587 | 34 351 | 519 |
