# Supplementary material for: Rabbit Microbiota Changes Throughout the Intestinal Tract
Source: Front Microbiol. 2018 Sep 13;9:2144. doi: 10.3389/fmicb.2018.02144 (PMC6146034; doi:10.3389/fmicb.2018.02144)
Supplement: Supplementary file 2 [file Table_2.DOCX]

**TABLE S2| OTUs differentially represented between fecal and cecal samples (*P*_FDR_ < 0.05)**

| **OUT ID and taxonomical assignment** | **Mean abundance (CSS OTU units) (SD)** | **Difference Cecum-Feces ± SE** | ***P_FDR_*** | ^a^**Discriminant sPLS-DA** |
| --- | --- | --- | --- | --- |
|  |  |  |  |  |
| **1110378**,Firmicutes;*Clostridia*;*Clostridiales*;*Ruminococcaceae*;*Oscillospira* | 2.648 (1.349) | 0.865 ± 0.198 | 0.000 | NO |
| **178839**,Firmicutes;*Clostridia*;*Clostridiales*;*Mogibacteriaceae* | 4.035 (0.744) | 0.387 ± 0.100 | 0.006 | NO |
| **207770**,Firmicutes;*Clostridia*;*Clostridiales*;*Ruminococcaceae*;*Ruminococcus* | 5.577 (1.869) | 0.755 ± 0.181 | 0.000 | YES |
| **210945**,Firmicutes;*Clostridia*;*Clostridiales*;*Clostridiaceae* | 3.761 (1.456) | 0.343 ± 0.123 | 0.050 | NO |
| **213671**,Bacteroidetes;*Bacteroidia*;*Bacteroidales*;*Rikenellaceae* | 1.317 (1.520) | 0.583 ± 0.166 | 0.006 | YES |
| **216941**,Verrucomicrobia;*Verrucomicrobiae*;*Verrucomicrobiales*;*Verrucomicrobiaceae*;*Akkermansia* | 7.113 (1.340) | 0.418 ± 0.110 | 0.006 | NO |
| **279340**,Firmicutes;*Clostridia*;*Clostridiales* | 1.374 (1.821) | 0.402 ± 0.173 | 0.017 | NO |
| **290079**,Firmicutes;*Clostridia*;*Clostridiales*;*Lachnospiraceae* | 1.759 (0.984) | 0.528 ± 0.201 | 0.021 | NO |
| **297503**,Firmicutes;*Clostridia*;*Clostridiales* | 3.852 (2.250) | 0.970 ± 0.201 | 0.000 | YES |
| **299422**,Verrucomicrobia;*Verrucomicrobiae*;*Verrucomicrobiales*;*Verrucomicrobiaceae*;*Akkermansia* | 5.210 (2.163) | 0.831 ± 0.179 | 0.000 | NO |
| **299902**,Firmicutes;*Clostridia*;*Clostridiales*;*Lachnospiraceae*;*Blautia* | 4.381 (1.781) | 0.852 ± 0.139 | 0.000 | NO |
| **303313**,Firmicutes;*Clostridia*;*Clostridiales*;*Lachnospiraceae*;*Blautia* | 1.608 (1.040) | 0.661 ± 0.187 | 0.000 | YES |
| **314586**,Firmicutes;*Clostridia*;*Clostridiales*;*Ruminococcaceae* | 3.710 (1.039) | 0.498 ± 0.146 | 0.021 | NO |
| **321135**,Firmicutes;*Clostridia*;*Clostridiales*;*Ruminococcaceae* | 2.505 (1.513) | 0.432 ± 0.145 | 0.015 | NO |
| **322258**,Firmicutes;*Clostridia*;*Clostridiales*;*Ruminococcaceae* | 2.917 (1.067) | 0.573 ± 0.162 | 0.006 | NO |
| **328083**,Firmicutes;*Clostridia*;*Clostridiales*;*Clostridiaceae*;*Clostridium* | 6.330 (1.059) | 0.576 ± 0.086 | 0.000 | YES |
| **330792**,Firmicutes;*Clostridia*;*Clostridiales* | 2.468 (1.296) | 0.313 ± 0.108 | 0.037 | NO |
| **342182**,Firmicutes;*Clostridia*;*Clostridiales* | 2.253 (2.401) | 0.514 ± 0.086 | 0.000 | NO |
| **352489**,Firmicutes;*Clostridia*;*Clostridiales*;*Lachnospiraceae* | 2.718 (1.055) | 0.734 ± 0.187 | 0.000 | NO |
| **385545**,Firmicutes;*Clostridia*;*Clostridiales* | 1.785 (1.709) | 0.588 ± 0.181 | 0.017 | YES |
| **423830**,Firmicutes;*Clostridia*;*Clostridiales* | 4.192 (1.568) | 0.770 ± 0.201 | 0.000 | NO |
| **527988**,Firmicutes;*Clostridia*;*Clostridiales*;*Eubacteriaceae*;*Anaerofustis* | 2.460 (1.108) | 0.409 ± 0.150 | 0.050 | NO |
| **576853**,Firmicutes;*Clostridia*;*Clostridiales* | 6.528 (1.110) | 0.424 ± 0.094 | 0.000 | NO |
| **580907**,Firmicutes;*Clostridia*;*Clostridiales*;*Ruminococcaceae* | 5.687 (0.945) | 0.648 ± 0.106 | 0.000 | YES |
| **589822**,Firmicutes;*Clostridia*;*Clostridiales*;*Lachnospiraceae*;*Blautia* | 2.533 (1.373) | 1.037 ± 0.169 | 0.000 | YES |
| **590015**,Firmicutes;*Clostridia*;*Clostridiales* | 3.221 (1.458) | 0.596 ± 0.143 | 0.006 | NO |
| **621649**,Firmicutes;*Clostridia*;*Clostridiales* | 9.088 (0.927) | 0.430 ± 0.086 | 0.000 | NO |
| **696563**,Firmicutes;*Clostridia*;*Clostridiales*;*Lachnospiraceae* | 3.773 (1.197) | 0.823 ± 0.142 | 0.000 | YES |
| **798164**,Firmicutes;*Clostridia*;*Clostridiales* | 3.114 (2.058) | 0.599 ± 0.188 | 0.021 | NO |
| **857827**,Bacteroidetes;*Bacteroidia*;*Bacteroidales* | 7.327 (2.595) | 0.503 ± 0.190 | 0.043 | YES |
| **NR0**,Firmicutes;*Clostridia*;*Clostridiales*;*Lachnospiraceae* | 5.491 (1.434) | 0.904 ± 0.114 | 0.000 | YES |
| **NR1**,Firmicutes;*Clostridia*;*Clostridiales*;*Ruminococcaceae*;*Ruminococcus* | 6.505 (0.789) | 0.336 ± 0.101 | 0.011 | NO |
| **NR104**,Firmicutes;*Clostridia*;*Clostridiales*;*Ruminococcaceae* | 8.199 (0.795) | 0.301 ± 0.094 | 0.011 | NO |
| **NR118**,Unknown | 4.575 (1.058) | 0.801 ± 0.167 | 0.000 | YES |
| **NR12**,Firmicutes;*Clostridia*;*Clostridiales* | 4.650 (1.058) | 1.706 ± 0.125 | 0.000 | YES |
| **NR123**,Firmicutes;*Clostridia*;*Clostridiales*;*Lachnospiraceae*;*Blautia* | 2.658 (1.735) | 0.658 ± 0.193 | 0.017 | YES |
| **NR128**,Firmicutes;*Clostridia*;*Clostridiales*;*Ruminococcaceae* | 1.514 (2.122) | 0.310 ± 0.108 | 0.017 | NO |
| **NR132**,Firmicutes;*Clostridia*;*Clostridiales*;*Lachnospiraceae* | 3.352 (2.047) | 0.728 ± 0.170 | 0.000 | YES |
| **NR138**,Unknown | 1.822 (1.066) | 0.716 ± 0.215 | 0.015 | YES |
| **NR152**,Firmicutes;*Clostridia*;*Clostridiales*;*Ruminococcaceae* | 10.895 (0.384) | 0.286 ± 0.080 | 0.000 | NO |
| **NR16**,Firmicutes;*Clostridia*;*Clostridiales* | 1.856 (1.948) | 0.531 ± 0.153 | 0.006 | NO |
| **NR167**,Firmicutes;*Clostridia*;*Clostridiales* | 2.074 (1.096) | 0.782 ± 0.216 | 0.000 | YES |
| **NR17**,Firmicutes;*Clostridia*;*Clostridiales* | 5.571 (0.897) | 0.776 ± 0.140 | 0.000 | YES |
| **NR174**,Firmicutes;*Clostridia*;*Clostridiales*;*Lachnospiraceae*;*Blautia* | 5.810 (1.084) | 1.010 ± 0.108 | 0.000 | YES |
| **NR176**,Unknown | 8.681 (0.978) | 0.948 ± 0.127 | 0.000 | YES |
| **NR181**,Firmicutes;*Clostridia*;*Clostridiales*;*Lachnospiraceae*;*Blautia* | 2.054 (1.498) | 0.720 ± 0.156 | 0.000 | YES |
| **NR197**,Firmicutes;*Clostridia*;*Clostridiales*;*Lachnospiraceae*;*Blautia* | 9.075 (1.003) | 0.583 ± 0.098 | 0.000 | YES |
| **NR199**,Firmicutes;*Clostridia*;*Clostridiales* | 2.258 (1.788) | 0.943 ± 0.314 | 0.017 | NO |
| **NR208**,Proteobacteria;*Betaproteobacteria* | 5.274 (0.831) | 0.801 ± 0.198 | 0.000 | YES |
| **NR212**,Firmicutes;*Clostridia*;*Clostridiales* | 4.328 (2.081) | 0.742 ± 0.198 | 0.006 | NO |
| **NR215**,Firmicutes;*Clostridia*;*Clostridiales*;*Veillonellaceae*;*Phascolarctobacterium* | 3.914 (3.498) | 0.550 ± 0.201 | 0.048 | NO |
| **NR218**,Firmicutes;*Clostridia*;*Clostridiales* | 2.083 (1.434) | 0.896 ± 0.163 | 0.000 | NO |
| **NR22**,Unknown | 2.966 (1.209) | 0.862 ± 0.228 | 0.017 | YES |
| **NR220**,Firmicutes;*Clostridia*;*Clostridiales* | 2.658 (2.171) | 0.985 ± 0.146 | 0.000 | NO |
| **NR224**,Firmicutes;*Clostridia*;*Clostridiales* | 4.542 (1.522) | 0.580 ± 0.117 | 0.000 | NO |
| **NR231**,Firmicutes;*Clostridia*;*Clostridiales*;*Lachnospiraceae* | 2.849 (2.131) | 0.423 ± 0.160 | 0.048 | NO |
| **NR234**,Firmicutes;*Clostridia*;*Clostridiales*;*Lachnospiraceae* | 3.051 (1.517) | 1.144 ± 0.147 | 0.000 | YES |
| **NR237**,Firmicutes;*Clostridia*;*Clostridiales*;*Clostridiaceae*;*Clostridium* | 1.640 (1.567) | 0.507 ± 0.171 | 0.021 | NO |
| **NR244**,Firmicutes;*Clostridia*;*Clostridiales*;*Lachnospiraceae*;*Blautia* | 2.894 (1.183) | 0.527 ± 0.197 | 0.048 | NO |
| **NR25**,Firmicutes;*Clostridia*;*Clostridiales*;*Lachnospiraceae* | 5.812 (1.026) | 0.411 ± 0.093 | 0.000 | NO |
| **NR259**,Unknown | 7.230 (2.010) | 1.038 ± 0.165 | 0.000 | YES |
| **NR274**,Firmicutes;*Clostridia*;*Clostridiales* | 3.637 (1.643) | 0.516 ± 0.169 | 0.028 | NO |
| **NR281**,Firmicutes;*Clostridia*;*Clostridiales*;*Lachnospiraceae* | 3.367 (1.217) | 0.448 ± 0.147 | 0.015 | YES |
| **NR286**,Firmicutes;*Clostridia*;*Clostridiales* | 4.013 (1.738) | 0.611 ± 0.156 | 0.011 | YES |
| **NR29**,Firmicutes;*Clostridia*;*Clostridiales* | 2.556 (3.770) | 0.731 ± 0.133 | 0.000 | YES |
| **NR297**,Unknown | 3.292 (1.028) | 0.794 ± 0.206 | 0.011 | YES |
| **NR308**,Firmicutes;*Clostridia*;*Clostridiales*;*Christensenellaceae* | 2.648 (1.459) | 0.407 ± 0.122 | 0.006 | NO |
| **NR316**,Firmicutes;*Clostridia*;*Clostridiales* | 2.251 (1.421) | 0.933 ± 0.288 | 0.031 | YES |
| **NR322**,Firmicutes;*Clostridia*;*Clostridiales*;*Mogibacteriaceae* | 3.246 (1.532) | 0.701 ± 0.130 | 0.000 | NO |
| **NR323**,Firmicutes;*Clostridia*;*Clostridiales*;*Lachnospiraceae*;*Blautia* | 1.991 (1.133) | 0.481 ± 0.146 | 0.017 | NO |
| **NR345**,Firmicutes;*Clostridia*;*Clostridiales*;*Lachnospiraceae* | 3.073 (1.222) | 0.415 ± 0.161 | 0.050 | NO |
| **NR352**,Firmicutes;*Clostridia*;*Clostridiales*;*Lachnospiraceae* | 6.028 (1.028) | 0.768 ± 0.108 | 0.000 | YES |
| **NR360**,Unknown | 2.963 (2.737) | 0.960 ± 0.189 | 0.000 | NO |
| **NR371**,Unknown | 5.485 (1.880) | 0.856 ± 0.203 | 0.006 | YES |
| **NR374**,Firmicutes;*Clostridia*;*Clostridiales*;*Lachnospiraceae*;*Blautia* | 1.912 (1.415) | 0.539 ± 0.164 | 0.024 | YES |
| **NR382**,Firmicutes;*Clostridia*;*Clostridiales* | 4.964 (2.243) | 0.513 ± 0.133 | 0.011 | NO |
| **NR383**,Firmicutes;*Clostridia*;*Clostridiales*;*Lachnospiraceae* | 3.189 (0.913) | 0.541 ± 0.175 | 0.017 | NO |
| **NR397**,Firmicutes;*Clostridia*;*Clostridiales* | 4.352 (1.673) | 0.381 ± 0.132 | 0.037 | NO |
| **NR4**,Firmicutes;*Clostridia*;*Clostridiales* | 2.970 (1.872) | 0.997 ± 0.262 | 0.011 | NO |
| **NR407**,Verrucomicrobia;*Verrucomicrobiae*;*Verrucomicrobiales*;*Verrucomicrobiaceae*;*Akkermansia* | 5.349 (1.766) | 0.635 ± 0.167 | 0.011 | NO |
| **NR417**,Firmicutes;*Clostridia*;*Clostridiales*;*Lachnospiraceae* | 4.225 (1.236) | 0.441 ± 0.156 | 0.043 | NO |
| **NR42**,Firmicutes;*Clostridia*;*Clostridiales*;*Clostridiaceae*;*Clostridium* | 6.506 (0.813) | 0.431 ± 0.097 | 0.000 | NO |
| **NR437**,Firmicutes;*Clostridia*;*Clostridiales* | 2.646 (1.755) | 0.566 ± 0.154 | 0.011 | NO |
| **NR443**,Firmicutes;*Clostridia*;*Clostridiales*;*Lachnospiraceae* | 3.385 (1.226) | 0.516 ± 0.167 | 0.017 | NO |
| **NR449**,Firmicutes;*Clostridia*;*Clostridiales*;*Lachnospiraceae* | 4.955 (1.343) | 0.588 ± 0.181 | 0.015 | NO |
| **NR451**,Tenericutes;*RF3*;*ML615J-28* | 2.079 (2.481) | 0.306 ± 0.123 | 0.039 | NO |
| **NR453**,Firmicutes;*Clostridia*;*Clostridiales*;*Lachnospiraceae* | 4.415 (0.856) | 0.545 ± 0.154 | 0.011 | NO |
| **NR455**,Actinobacteria;*Coriobacteriia*;*Coriobacteriales* | 3.385 (1.272) | 0.617 ± 0.135 | 0.000 | YES |
| **NR457**,Firmicutes;*Clostridia*;*Clostridiales* | 2.558 (2.209) | 0.571 ± 0.153 | 0.000 | NO |
| **NR50**,Actinobacteria;*Coriobacteriia*;*Coriobacteriales*;*Coriobacteriaceae* | 5.889 (0.823) | 0.887 ± 0.132 | 0.000 | YES |
| **NR54**,Firmicutes;*Clostridia*;*Clostridiales* | 2.457 (1.575) | 0.380 ± 0.145 | 0.048 | NO |
| **NR6**,Firmicutes;*Clostridia*;*Clostridiales*;*Ruminococcaceae* | 3.433 (1.406) | 0.475 ± 0.154 | 0.037 | NO |
| **NR7**,Firmicutes;*Clostridia*;*Clostridiales*;*Clostridiaceae*;*Clostridium* | 4.916 (1.050) | 0.594 ± 0.097 | 0.000 | YES |
| **NR71**,Unknown | 2.842 (1.566) | 1.075 ± 0.258 | 0.000 | YES |
| **NR76**,Firmicutes;*Clostridia*;*Clostridiales* | 2.242 (1.293) | 0.404 ± 0.150 | 0.028 | YES |
| **NR86**,Unknown | 5.277 (1.211) | 0.664 ± 0.236 | 0.031 | NO |
| **NR96**,Verrucomicrobia;*Verrucomicrobiae*;*Verrucomicrobiales*;*Verrucomicrobiaceae*;*Akkermansia* | 3.982 (1.742) | 0.728 ± 0.173 | 0.006 | YES |
| **1108356**,Tenericutes;*Mollicutes*;*RF39* | 4.885 (0.863) | -0.520 ± 0.157 | 0.017 | NO |
| **157802**,Firmicutes;*Clostridia*;*Clostridiales* | 3.029 (1.465) | -0.536 ± 0.111 | 0.000 | NO |
| **173245**,Firmicutes;*Clostridia*;*Clostridiales* | 5.881 (1.618) | -0.748 ± 0.166 | 0.006 | NO |
| **197832**,Firmicutes;*Clostridia*;*Clostridiales*;*Lachnospiraceae*;*Coprococcus* | 3.295 (3.191) | -1.208 ± 0.357 | 0.011 | NO |
| **206151**,Firmicutes;*Clostridia*;*Clostridiales*;*Lachnospiraceae*;*Coprococcus* | 5.146 (1.754) | -1.092 ± 0.266 | 0.000 | NO |
| **208769**,Firmicutes;*Clostridia*;*Clostridiales*;*Ruminococcaceae* | 4.523 (1.031) | -0.630 ± 0.183 | 0.015 | NO |
| **209492**,Firmicutes;*Clostridia*;*Clostridiales*;*Ruminococcaceae* | 4.083 (0.941) | -0.442 ± 0.161 | 0.050 | NO |
| **209524**,Firmicutes;*Clostridia*;*Clostridiales*;*Lachnospiraceae*;*Coprococcus* | 4.180 (2.710) | -0.815 ± 0.206 | 0.000 | YES |
| **210867**,Firmicutes;*Clostridia*;*Clostridiales* | 1.571 (2.383) | -0.664 ± 0.252 | 0.039 | NO |
| **210895**,Firmicutes;*Clostridia*;*Clostridiales* | 5.284 (1.511) | -0.514 ± 0.105 | 0.000 | NO |
| **258404**,Firmicutes;*Clostridia*;*Clostridiales*;*Ruminococcaceae*;*Ruminococcus* | 1.934 (1.883) | -0.541 ± 0.185 | 0.039 | NO |
| **258980**,Firmicutes;*Clostridia*;*Clostridiales* | 2.062 (1.775) | -0.441 ± 0.148 | 0.024 | NO |
| **261966**,Firmicutes;*Clostridia*;*Clostridiales* | 4.416 (1.878) | -0.815 ± 0.274 | 0.034 | NO |
| **267220**,Bacteroidetes;*Bacteroidia*;*Bacteroidales*;*S24-7* | 3.001 (2.285) | -0.828 ± 0.190 | 0.000 | NO |
| **269386**,Bacteroidetes;*Cyanobacteria*;*4C0d-2*;*YS2* | 1.453 (1.619) | -0.948 ± 0.281 | 0.011 | NO |
| **275194**,Firmicutes;*Clostridia*;*Clostridiales* | 3.675 (1.664) | -0.597 ± 0.143 | 0.000 | NO |
| **279048**,Cyanobacteria;*4C0d-2*;*YS2* | 1.617 (1.714) | -1.115 ± 0.282 | 0.006 | NO |
| **279179**,Tenericutes;*Mollicutes*;*RF39* | 2.974 (1.906) | -0.704 ± 0.195 | 0.015 | NO |
| **288193**,Firmicutes;*Clostridia*;*Clostridiales* | 4.272 (1.229) | -0.712 ± 0.157 | 0.000 | YES |
| **289538**,Firmicutes;*Clostridia*;*Clostridiales* | 2.647 (1.282) | -0.904 ± 0.194 | 0.006 | NO |
| **313524**,Firmicutes;*Clostridia*;*Clostridiales*;*Ruminococcaceae* | 2.081 (1.101) | -0.815 ± 0.216 | 0.011 | YES |
| **314029**,Firmicutes;*Clostridia*;*Clostridiales* | 2.874 (2.558) | -0.370 ± 0.133 | 0.034 | NO |
| **326013**,Firmicutes;*Clostridia*;*Clostridiales* | 5.019 (1.803) | -0.875 ± 0.156 | 0.000 | YES |
| **333768**,Firmicutes;*Clostridia*;*Clostridiales*;*Ruminococcaceae*;*Oscillospira* | 3.239 (0.993) | -0.687 ± 0.195 | 0.006 | NO |
| **337724**,Bacteroidetes;*Bacteroidia*;*Bacteroidales*;*S24-7* | 1.560 (2.540) | -0.240 ± 0.085 | 0.021 | NO |
| **341902**,Firmicutes;*Clostridia*;*Clostridiales*;*Ruminococcaceae*;*Oscillospira* | 1.960 (0.851) | -0.807 ± 0.216 | 0.000 | YES |
| **345556**,Firmicutes;*Clostridia*;*Clostridiales* | 9.442 (0.849) | -0.503 ± 0.201 | 0.020 | YES |
| **355312**,Firmicutes;*Clostridia*;*Clostridiales* | 2.019 (1.751) | -0.730 ± 0.210 | 0.011 | NO |
| **355494**,Firmicutes;*Clostridia*;*Clostridiales* | 3.751 (1.175) | -0.733 ± 0.151 | 0.000 | YES |
| **408513**,Firmicutes;*Clostridia*;*Clostridiales*;*Ruminococcaceae*;*Oscillospira* | 2.535 (0.938) | -0.952 ± 0.187 | 0.000 | YES |
| **422283**,Firmicutes;*Clostridia*;*Clostridiales*;*Ruminococcaceae*;*Oscillospira* | 6.000 (0.675) | -0.467 ± 0.119 | 0.017 | YES |
| **4343981**,Tenericutes;*Mollicutes*;*RF39* | 4.024 (0.901) | -0.384 ± 0.128 | 0.037 | NO |
| **443620**,Firmicutes;*Clostridia*;*Clostridiales*;*Ruminococcaceae*;*Oscillospira* | 3.316 (1.081) | -0.766 ± 0.195 | 0.000 | YES |
| **4443094**,Firmicutes;*Clostridia*;*Clostridiales* | 2.057 (1.167) | -0.603 ± 0.224 | 0.050 | NO |
| **514061**,Tenericutes;*Mollicutes*;*RF39* | 5.081 (1.022) | -0.596 ± 0.122 | 0.000 | YES |
| **523099**,Firmicutes;*Clostridia*;*Clostridiales* | 2.510 (1.035) | -0.478 ± 0.222 | 0.032 | YES |
| **528071**,Firmicutes;*Clostridia*;*Clostridiales* | 1.551 (0.981) | -0.630 ± 0.205 | 0.017 | NO |
| **542830**,Cyanobacteria;*4C0d-2*;*YS2* | 2.340 (1.800) | -1.313 ± 0.309 | 0.011 | NO |
| **550894**,Cyanobacteria;*4C0d-2*;*YS2* | 3.651 (1.871) | -1.713 ± 0.219 | 0.000 | YES |
| **559149**,Cyanobacteria;*4C0d-2*;*YS2* | 2.575 (1.424) | -0.652 ± 0.223 | 0.028 | NO |
| **571111**,Firmicutes;*Clostridia*;*Clostridiales*;*Ruminococcaceae*;*Ruminococcus* | 2.314 (1.773) | -0.675 ± 0.199 | 0.006 | NO |
| **581388**,Cyanobacteria;*4C0d-2*;*YS2* | 3.847 (1.935) | -2.034 ± 0.173 | 0.000 | YES |
| **584263**,Firmicutes;*Clostridia*;*Clostridiales* | 4.379 (1.568) | -0.541 ± 0.174 | 0.015 | NO |
| **589410**,Cyanobacteria;*4C0d-2*;*YS2* | 1.649 (1.468) | -1.544 ± 0.190 | 0.000 | YES |
| **640999**,Firmicutes;*Clostridia*;*Clostridiales* | 5.065 (1.527) | -0.576 ± 0.171 | 0.015 | NO |
| **646499**,Firmicutes;*Clostridia*;*Clostridiales* | 3.368 (1.567) | -1.009 ± 0.224 | 0.006 | YES |
| **715152**,Firmicutes;*Clostridia*;*Clostridiales*;*Ruminococcaceae* | 3.609 (1.625) | -0.418 ± 0.204 | 0.063 | YES |
| **769075**,Firmicutes;*Clostridia*;*Clostridiales* | 2.980 (1.151) | -0.830 ± 0.202 | 0.000 | NO |
| **772972**,Firmicutes;*Clostridia*;*Clostridiales* | 4.768 (1.378) | -0.563 ± 0.163 | 0.011 | NO |
| **786646**,Firmicutes;*Clostridia*;*Clostridiales* | 1.614 (1.073) | -0.545 ± 0.206 | 0.034 | YES |
| **NR102**,Firmicutes;*Clostridia*;*Clostridiales*;*Ruminococcaceae* | 8.501 (0.671) | -0.302 ± 0.126 | 0.050 | NO |
| **NR108**,Firmicutes;*Clostridia*;*Clostridiales*;*Ruminococcaceae* | 6.741 (2.712) | -0.382 ± 0.168 | 0.050 | NO |
| **NR116**,Firmicutes;*Clostridia*;*Clostridiales* | 3.599 (2.915) | -0.608 ± 0.175 | 0.000 | NO |
| **NR117**,Firmicutes;*Clostridia*;*Clostridiales* | 4.705 (1.478) | -0.845 ± 0.169 | 0.000 | NO |
| **NR142**,Proteobacteria;*Alphaproteobacteria* | 3.271 (1.553) | -1.075 ± 0.220 | 0.000 | YES |
| **NR163**,Tenericutes;*Mollicutes*;*RF39* | 3.366 (1.355) | -0.540 ± 0.176 | 0.024 | NO |
| **NR173**,Firmicutes;*Clostridia*;*Clostridiales*;*Ruminococcaceae* | 4.582 (0.864) | -0.798 ± 0.135 | 0.000 | YES |
| **NR177**,Bacteroidetes;*Bacteroidia*;*Bacteroidales*;*S24-7* | 2.379 (3.298) | -0.416 ± 0.139 | 0.006 | NO |
| **NR19**,Firmicutes;*Clostridia*;*Clostridiales* | 4.534 (1.063) | -0.585 ± 0.142 | 0.000 | YES |
| **NR194**,Tenericutes;*Mollicutes*;*RF39* | 5.479 (0.968) | -0.513 ± 0.152 | 0.017 | NO |
| **NR211**,Tenericutes;*Mollicutes*;*RF39* | 2.331 (1.948) | -0.343 ± 0.119 | 0.017 | NO |
| **NR23**,Firmicutes;*Clostridia*;*Clostridiales*;*Ruminococcaceae* | 4.874 (0.777) | -0.526 ± 0.167 | 0.031 | YES |
| **NR242**,Firmicutes;*Clostridia*;*Clostridiales* | 4.016 (2.260) | -0.677 ± 0.183 | 0.015 | NO |
| **NR247**,Firmicutes;*Clostridia*;*Clostridiales* | 1.782 (1.195) | -0.788 ± 0.191 | 0.006 | YES |
| **NR256**,Tenericutes;*Mollicutes*;*RF39* | 2.871 (1.866) | -0.653 ± 0.247 | 0.037 | NO |
| **NR28**,Firmicutes;*Clostridia*;*Clostridiales*;*Ruminococcaceae* | 7.464 (1.893) | -1.860 ± 0.180 | 0.000 | YES |
| **NR294**,Firmicutes;*Clostridia*;*Clostridiales*;*Ruminococcaceae*;*Oscillospira* | 6.143 (1.036) | -0.953 ± 0.199 | 0.000 | YES |
| **NR295**,Bacteroidetes;*Bacteroidia*;*Bacteroidales*;*S24-7* | 2.556 (3.770) | -0.267 ± 0.097 | 0.037 | NO |
| **NR307**,Firmicutes;*Clostridia*;*Clostridiales*;*Ruminococcaceae* | 5.790 (0.696) | -0.428 ± 0.131 | 0.011 | NO |
| **NR310**,Firmicutes;*Clostridia*;*Clostridiales* | 4.732 (2.305) | -0.957 ± 0.167 | 0.000 | NO |
| **NR32**,Firmicutes;*Clostridia*;*Clostridiales*;*Ruminococcaceae*;*Ruminococcus* | 2.656 (1.504) | -0.571 ± 0.230 | 0.039 | NO |
| **NR338**,Firmicutes;*Clostridia*;*Clostridiales* | 4.746 (1.384) | -0.825 ± 0.149 | 0.000 | YES |
| **NR339**,Firmicutes;*Clostridia*;*Clostridiales* | 4.439 (1.403) | -0.721 ± 0.182 | 0.000 | NO |
| **NR349**,Firmicutes;*Clostridia*;*Clostridiales*;*Ruminococcaceae*;*Ruminococcus* | 3.281 (1.246) | -0.829 ± 0.259 | 0.021 | NO |
| **NR361**,Firmicutes;*Clostridia*;*Clostridiales* | 3.909 (1.022) | -0.767 ± 0.150 | 0.000 | YES |
| **NR37**,Firmicutes;*Clostridia*;*Clostridiales*;*Ruminococcaceae*;*Oscillospira* | 2.407 (1.276) | -0.853 ± 0.267 | 0.006 | NO |
| **NR395**,Firmicutes;*Clostridia*;*Clostridiales* | 6.412 (1.790) | -0.901 ± 0.243 | 0.006 | NO |
| **NR410**,Firmicutes;*Clostridia*;*Clostridiales*;*Ruminococcaceae* | 3.770 (1.750) | -0.363 ± 0.131 | 0.021 | YES |
| **NR411**,Proteobacteria;*Alphaproteobacteria*;*RF32* | 1.990 (1.551) | -1.214 ± 0.255 | 0.000 | YES |
| **NR414**,Firmicutes;*Clostridia*;*Clostridiales* | 4.655 (2.255) | -0.628 ± 0.222 | 0.048 | NO |
| **NR423**,Firmicutes;*Clostridia*;*Clostridiales* | 3.846 (1.439) | -0.921 ± 0.175 | 0.000 | NO |
| **NR448**,Tenericutes;*Mollicutes*;*RF39* | 4.128 (0.975) | -0.542 ± 0.141 | 0.006 | NO |
| **NR456**,Firmicutes;*Clostridia*;*Clostridiales*;*Ruminococcaceae*;*Ruminococcus* | 1.022 (1.316) | -0.784 ± 0.192 | 0.011 | NO |
| **NR461**,Tenericutes;*Mollicutes*;*RF39* | 2.820 (1.325) | -0.384 ± 0.110 | 0.017 | NO |
| **NR57**,Firmicutes;*Clostridia*;*Clostridiales*;*Ruminococcaceae* | 3.603 (2.298) | -2.503 ± 0.311 | 0.000 | NO |
| **NR60**,Firmicutes;*Clostridia*;*Clostridiales* | 2.944 (2.283) | -2.247 ± 0.201 | 0.000 | NO |
| **NR87**,Firmicutes;*Clostridia*;*Clostridiales*;*Lachnospiraceae*;*Coprococcus* | 3.547 (2.668) | -0.480 ± 0.129 | 0.021 | NO |

^a^Last column indicates whether the OTU belongs to component 1 of sPLS-DA.
