# Supplementary material for: Rabbit Microbiota Changes Throughout the Intestinal Tract
Source: Front Microbiol. 2018 Sep 13;9:2144. doi: 10.3389/fmicb.2018.02144 (PMC6146034; doi:10.3389/fmicb.2018.02144)
Supplement: Supplementary file 3 [file Data_Sheet_1.docx]

**Supplementary Data Sheet 1.| Representative sequences of the 10 OTUs most differentially represented between fecal and cecal samples.**

>**NR57**

GTGTCAGCCGCCGCGGTAATACGTAGGGAGCGAGCGTTGTCCGGAATTACTGGGTGTAAAGGGAGTGTAGGCGGGACTGTAAGTCAGATGTGAAATGTAGGGGCTCAACCCCTGCCCTGCATTTGAAACTGTAGTTCTTGAGTGAAGTAGAGGTAAGCGGAATTCCTAGTGTAGCGGTGAAATGCGTAGATATTAGGAGGAACATCAGTGGCGAAGGCGGCTTACTGGGCTTTTACTGACGCTGAGGCTCGAAAGCGTGGGGAGCAAACAGGATTAGATACCCTGGTAGTCCACGCTGTAAACGATGATCACTAGGTGTGGGGGGACTGACCCCTTCCGTGCCGCAGTTAACACAATAAGTGATCCACCTGGGGAGTACGGCCGCAAGGCTGAAACTCAAAGAAATTGACGG

>**NR60**

GTGTCAGCAGCCGCGGTAATACGTAGGGGGCAAGCGTTATCCGGATTTACTGGGTGTAAAGGGAGCGTAGGTGGCGGTGCAAGTCAGAAGTGAAATGCCGGGGCTCAACCCCGGAGCTGCTTTTGTAACTGCACAGCTGGAGTGCAGGAGGGGTAAGCGGAATTCCTAGTGTAGCGGTGAAATGCGTAGATATTAGGAGGAACACCGGTGGCGAAGGCGGCTTACTGGACTGTAACTGACACTGAGGCTCGAAAGCGTGGGGAGCAAACAGGATTAGATACCCTGGTAGTCCACGCCGTAAACGATGAATACTAGGTGTCGGGGAGCATCAGCTCTTCGGTGCCGCAGCCAACGCAATAAGTATTCCACCTGGGGAGTACGTTCGCAAGAATGAAACTCAAATGAATTGGCGG

>**581388**

GTGTCAGCAGCCGCGGTAATACGGGGGGTGCAAGCGTTGTCCGGAATCATTGGGCGTAAAGCGTTCGTAGGCGGCATGCCAAGTCTGGTGTTAAATCCCGGGGCTCAACTCCGGTCAAGCATTGGATACTGGTAAGCTAGAATGTGGTAGAGGTTAAGGGAATTCCTGGTGTAGCGGTGAAATGCGTAGATATCAGGAGGAACACCGGTGGCGTAAGCGCTTAACTGGGCCATAATTGACGCTGAGGAACGAAAGCCGGGGTAGCAAATGGGATTAGATACCCCAGTAGTCCCGGCTGTAAACGATGGATACTAGGTGTTGCGGGTATCGACCCCTGCAGTGCCGCAGCCAACGCGATAAGTATCCCGCCTGGGGAGTACGCACGCAAGTGTGAAACTCAAAGAAATTGACGG

>**NR28**

GTGTCAGCAGCCGCGGTAATACGTAGGGAGCGAGCGTTGTCCGGAATTACTGGGTGTAAAGGGAGCGTAGGCGGGGTTGCAAGTCAGATGTGAAAAGTAGGGGCTTAACCCCTGAACTGCATTTGAAACTGTAATTCTTGAGTGAAGTAGAGGTAAGCGGAATTCCTAGTGTAGCGGTGAAATGCGTAGATATTAGGAGGAACATCAGTGGCGAAGGCGGCTTACTGGGCTTTAACTGACGCTGAGGCTCGAAAGCGTGGGGAGCAAACAGGATTAGATACCCTGGTAGTCCACGCTGTAAACGATGATCACTAGGTGTGGGGGGATAAGGACCCTTCCGTGCCGCAGTTAACACAATAAGTGATCCACCTGGGGAGTACGGTCGCAAGGCTGAAACTCAAATAAATTGACGG

>**550894**

GTGTCAGCAGCCGCGGTAATACGGGGGGTGCAAGCGTTGTCCGGAATCATTGGGCGTAAAGCGTTCGTAGGCGGCATGCCAAGTCTGGTGTTAAATCCCGGGGCTCAACTCCGGTCAAGCATTGGATACTGGTAAGCTAGAATGTGGTAGAGGTTAAGGGAATTCCTGGTGTAGCGGTGAAATGCGTAGATATCAGGAGGAACACCGGTGGCGTAAGCGCTTAACTGGGCCATAATTGACGCTGAGGAACGAAAGCCGGGGTAGCAAATGGGATTAGATACCCCAGTAGTCCCGGCTGTAAACGATGGATACTAGGTGTTGCGGGTATCGACCCCTGCAGTGCCGCAGCCAACGCGATAAGTATCCCGCCTGGGGAGTACGCACGCAAGTGTGAAACTCAAATAAATTGACGG

>**NR12**

GTGTCAGCAGCCGCGGTAATACGTAGGGGGCAAGCGTTATCCGGATTTACTGGGTGTAATGGGAGCGCAGGCGGCGATGCAAGCCAGAAGTGAAAACCCGGGGCCCAACCCCGCGGATTGCTTTTGGAACTGTGTTGCTGGAGTGCAGGAGAGGCAAGCGGAATTCCTGGTGTAGCGGTGAAATGCGTAGATATCAGGAGGAACACCGGTGGTGAAGGCGGCTTGCTGGACTGTAACTGACGCTGAGGCTCGAAAGCGTGGGGAGCAAACAGGATTAGATACCCTGGTAGTCCACGCGGTAAACGATGAATACTAGGTGTCGGTGTGCAAAGCGCATCGGTGCCGCAGCTAACGCAGTAAGTATTCCACCTGGGGAGTACGTTCGCAAGAATGAAACTCAAATAAATTGACGG

>**589410**

GTGTCAGCAGCCGCGGTAATACGGGGGGTGCAAGCGTTGTCCGGAATCATTGGGCGTAAAGCGTTCGTAGGCGGTATGTCAAGTCTGGTGTTAAATCCCGGGGCTTAACTCCGGTCCAGCATTGGATACTGGCAAACTAGAATGTGGTAGAGGTAAAGGGAATTCCTGGTGTAGCGGTGAAATGCGTAGATATCAGGAGGAACACCGGTGGCGTAAGCGCTTTACTGGGCCATAATTGACGCTGAGGAACGAAAGCCGGGGGAGCAAATGGGATTAGATACCCCAGTAGTCCCGGCCGTAAACGATGGATACTAGGTGTTGCGGGTATCGACCCCTGCAGTGCCGCAGCCAACGCGATAAGTATCCCGCCTGGGGAGTACGCACGCAAGTGTGAAACTCAAAGAAATTGACGG

>**542830**

GTGTCAGCAGCCGCGGTAATACGGGGGGTGCAAGCGTTGTCCGGAATCATTGGGCGTAAAGCGTTCGTAGGCGGCATGCCAAGTCTGGTGTTAAATCCCGGGGCTCAACTCCGGTCAAGCATTGGATACTGGTAAGCTAGAATGTGGTAGAGGTTAAGGGAATTCCTGGTGTAGCGGTGAAATGCGTAGATATCAGGAGGAACACCGGTGGCGTAAGCGCTTAACTGGGCCATAATTGACGCTGAGGAACGAAAGCCGGGGTAGCAAATGGGATTAGATCCCCCAGTAGTCCCGGCTGTAAACGATGGATACTAGGTGTTGCGGGTATCGACCCCTGCAGTGCCGCAGCCAACGCGATAAGTATCCCGCCTGGGGAGTACGCACGCAAGTGTGAAACTCAAATAAATTGGCGG

>**NR411**

GTGCCAGCAGCCGCGGTAATACGAAGGGTGCGAGCGTTGTTCGGAATTACTGGGCGTAAAGGGTGAGTAGGCGGTTTAGTAAGATAGCGGTGAAATGCCAGAGCTTAACTTTGGAATTGCCGTTATAACTATTAAGCTAGAGTGACAGAGAGGATATTGGAATACCCAGTGTAGAGGTGAAATTCGTAGATATTGGGTAGAACACCGGTGGCGAAGGCGAGTATCTGGCTGTAGACTGACGCTGAGGCACGAAAGCATGGGGATCAAACAGGATTAGATACCCTGGTAGTCCATGCTGTAAACGATGAATGCTAGTTGTTGGTAGGGATCAGTGACGAAGCAAACGCGATAAGCATTCCGCCTGGGGAGTACGGCCGCAAGGTTAAAACTCAAATAAATTGACGG

>**197832**

GTGTCAGCAGCCGCGGTAATACGTATGGTGCAAGCGTTATCCGGATTTACTGGGTGTAAAGGGTGCGTAGGTGGTGAGACAAGTCTGAAGTGAAAATCCGGGGCTCAACCCCGGAACTGCTTTGGAAACTGCCTGACTGGAGTACAGGAGAGGTAAGTGGAATTCCTAGTGTAGCGGTGAAATGCGTAGATATTAGGAGGAACACCAGTGGCGAAGGCGACTTACTGGACTGTAACTGACACTGAGGCACGAAAGCGTGGGGAGCAAACAGGATTAGATACCCTGGTAGTCCACGCCGTAAACGATGAATACTAGGTGTCGGGGCCCAAAGGGCTTCGGTGCCGCAGCAAACGCAATAAGTATTCCACCTGGGGAGTACGTTCGCAAGAATGAAACTCAAAGAAATTGACGG
